# Second Graders’ Grapho-Motor Skill Learning and Verbal Learning: The Effects of Socio-Educational Factors

**DOI:** 10.3389/fpsyg.2021.687207

**Published:** 2021-10-12

**Authors:** Chagit Hollander, Esther Adi-Japha

**Affiliations:** ^1^School of Education, Bar-Ilan University, Ramat Gan, Israel; ^2^The Leslie and Susan Gonda (Goldschmied) Multidisciplinary Brain Research Center, Bar-Ilan University, Ramat Gan, Israel

**Keywords:** developmental dyslexia, procedural-learning, low-SES children, maternal education level, grapho-motor learning, implicit-memory, verbal-learning, literacy

## Abstract

**Introduction:** Children from low socioeconomic status (SES) families, and in particular, those with a lower level of maternal education, show lower fine-motor skills and lower vocabulary scores than their SES peers whose mothers have a higher level of education. Furthermore, low SES children frequently have difficulties in reading and spelling. These difficulties are attributed to deficits in the acquisition of skills through practice, such as those required for developing visual-motor routines, alongside deficits in the intentional acquisition of knowledge, such as those required in verbal learning. The aim of the current study was to test the effect of two background factors: low maternal education (ME) and risk of reading and spelling difficulties on practice-dependent learning of a motor task and intentional learning of a verbal task in second graders from low SES families.

**Methods:** In 2016/17, 134 low-SES second graders with higher and lower ME (95 typical learners and 39 with reading and spelling difficulties) were assessed with (a) the Invented Letter Task (ILT; a grapho-motor skill learning task) across five time-points (initial- and end-training Day 1; initial- and end-training Day 2; and 2-weeks post-training), as well as an ILT transfer task; and (b) The Rey Auditory Verbal Learning Test (RAVLT; an intentional word-learning task in which a word list is read to children for five learning trials and is recalled 20 min later).

**Findings:** Lower ME was associated with surplus segments in the performance of the motor task and its transfer to a novel condition as well as with lower recall on the verbal task, but not with the learning of both the motor and the verbal task. Having reading and spelling difficulties affected motor-task accuracy and also the way children learned the task, as evidenced by surplus segments at the beginning of Day 2, which were reduced with further practice.

**Conclusion:** Low ME affected overall performance level. Reading and spelling difficulties resulted in atypical learning of the motor task. Future research on practice-dependent learning in the context of children coming from low SES families should focus on subgroups within this heterogeneous population.

## Introduction

Motor skills and verbal knowledge are two core domains of development learned by children following repeated experiences. Children from low socioeconomic status (SES) families repeatedly demonstrate diminished educational attainment, language, and fine motor skills, thus widening the achievement gap between higher and lower SES groups across school years (Brooks-Gunn and Duncan, 1997; Grissmer et al., 2010; Roy et al., 2014). Evidence suggests, however, that these children’s learning may be further moderated under specific circumstances. For example, children from low-SES families in which maternal education (ME) is low were reported to have even fewer learning experiences (Bradley et al., 2001; Bradley and Corwyn, 2002; Milne and Plourde, 2006; Elliott, 2020). Furthermore, children from low SES families who also experience reading and spelling difficulties may show poorer fine motor skills and verbal memory than their low SES peers, as these domains are commonly impaired in children with reading and spelling difficulties (Scarborough, 1998; Costa et al., 2018). Although motor skills and verbal knowledge have been studied in children from low SES families, there is a paucity of research of subgroups within this heterogeneous population. In particular, it is not clear whether lower ME and the presence of reading and spelling difficulties may result in even lower performance levels. Furthermore, it is not known whether lower performance levels in low SES children, and in particular, in those with low ME or reading and spelling difficulties, may be associated with atypical learning and retention of new information in memory following a practice experience, possibly due to initial lower performance levels that may hamper learning (Bardid et al., 2013; van Tetering et al., 2018). These questions are of particular importance when planning school curricula and intervention programs for the enhancement of basic skills in children coming from low SES families.

A central tenet in neurobehavioral science is that long-term memories are sub-served by separate and distinct systems: the procedural and the declarative. The procedural system facilitates the acquisition of skills and routines through numerous repeated experiences (*how to* knowledge) and necessitate a critical number of repetitions and amount time. Once acquired, they are lasting (Squire and Dede, 2015). The declarative system is dedicated to the learning, remembering, and use of facts and events (*what* knowledge). Learning is intentional and requires one or few repetitions. Knowledge is rapidly acquired, although it may also be rapidly forgotten. Research in recent years has focused on the neural basis of learning with reference to the specific domain of learning (Raviv and Arnon, 2018). While motor proficiency, including that required for reading and spelling, such as the parsing of discrete elements and horizontal progression between elements, is acquired through repeated practice (Julius et al., 2016), verbal learning, e.g., of words, is considered more intentional (Hamrick et al., 2018). It has been suggested that low SES is strongly associated with deficits in the intentional acquisition of knowledge through the declarative memory system and with the linguistic domain (Farah, 2017), but not with the acquisition of practice-dependent skills (Leonard et al., 2015). The latter hypothesis, however, has not been investigated in children, and particularly in relation to motor skill learning.

### Motor Skill Learning

Motor skill learning is the process by which movements are executed more quickly and accurately with practice (Willingham, 1998). The study of motor skill learning differentiates between the overall level of performance and performance change across the different learning phases and transfer conditions. Skill acquisition initially develops relatively quickly across different experimental paradigms (i.e., rapid improvements measured over the course of a single training session) and later slows as further gains develop incrementally over multiple practice sessions, until performance reaches nearly asymptotic levels. The progression from fast to slow motor learning is thought to depend on appropriate consolidation processes, defined as the progressive stabilization of a recently acquired memory (Dudai, 2004). Memory-consolidation processes are triggered by the training experience but require time (say, 24 h) to reach successful completion. Retention is tested at a delay of days or weeks. Performance measures commonly include speed and accuracy. The time trajectory of motor skill learning has been mainly studied in adults and more recently, in children (Dorfberger et al., 2007; Adi-Japha et al., 2008; Savion-Lemieux et al., 2009; Julius and Adi-Japha, 2015).

### Verbal Learning

Language-processing difficulties are assessed using several tasks, including story recall, paired-association learning, and list learning. List-learning tasks test the ability to intentionally learn and memorize a list of unrelated words after repeated practice, enabling the study of factors that affect practice-dependent learning and retention in the relevant domain. Young children are not expected to remember long word lists. Only at older ages does reading typically involve sentences and texts of considerable length; at that stage, high achievement may increasingly depend on the child’s ability to retain material in memory as it is being read in order to facilitate the syntactic and semantic analyses that are necessary for comprehension (Scarborough, 1998).

### Low Socioeconomic Status and Maternal Education

Low SES families comprise a heterogeneous population. SES is commonly indexed by several variables such as parental education, income, and profession. However, a plethora of studies concerning SES differences – even within the low SES population – refer to parental education level as an additional index of interest (Ganzach, 2000; Korat et al., 2007; Fluss et al., 2009; Zadeh et al., 2010; Yoshikawa et al., 2012; Hoff, 2013; Gullick et al., 2016; Mendive et al., 2017). Similarly, the current study indexed lower/higher ME levels within a low-SES sample.

### Motor Skill Learning in Children From Low Socioeconomic Status Families and the Effect of Maternal Education

The level of motor skills, in particular of grapho-motor and visual-motor skills, is lower among low-SES children (Dinehart and Manfra, 2013; Julius et al., 2016; Africa and van Deventer, 2017). Regarding the effect of ME, previous studies found a tendency for better fine and gross motor skills among children of better-educated parents than among those of less educated parents. A recent study reported that only the mother’s (but not the father’s) education level was related to fine and gross motor skill development. Children of mothers who had post-high school degrees were assessed as having a significantly higher level of skill development, compared to mothers who had high school diplomas or had not completed high school (Valadi and Gabbard, 2020). Although we were not able to find studies assessing the effect of parental education on motor achievements in children coming from low-SES families, a study conducted in Egypt found significant delays, especially in fine motor skills, which were related to parental education level (El Elella et al., 2017). Across studies, environmental affordances were suggested as explaining lower motor skills.

Our literature search yielded no studies that assessed the effect of SES on the time-course of practicing novel motor skills, but we were able to find a few motor intervention studies that focused on the effect of SES. These intervention studies presented mixed findings for the effect of SES on practice outcomes (De Bourdeaudhuij et al., 2011; Bardid et al., 2013; Birnbaum et al., 2017), with some studies reporting narrowing of gaps, and others reporting widening of gaps or their remaining effect, even within the same study. One reason for the mixed effect may be the heterogeneity of the low-SES population, which may affect initial performance level as well as practice gains (Bardid et al., 2013).

### Verbal Learning in Children From Low Socioeconomic Status Families and the Effect of Maternal Education

Maternal education is strongly related to the richness of children’s verbal input and has been shown to be the most important SES component in predicting language development and children’s literacy development (Hoff, 2003, 2013). Milne and Plourde (2006) observed six low-SES families whose children received free or reduced-price lunches and had high achievements. They found that parents with relatively stronger education provided their children with stimulating and literacy-rich opportunities by, for example, making reading materials available at home, and that their children had higher educational achievements. Milne and Plourde concluded that factors such as income may not necessarily be the best predictors of children’s achievements. Fluss et al. (2009), who also studied a low–medium SES population as defined by the national allocation of supplementary educational resources to disadvantaged school districts, suggested that higher ME is likely to be associated with a higher frequency of promoting literacy activities and more developed linguistic skills, further stressing the effect of ME on children’s practice opportunities.

Although verbal processing is a core deficit in low-SES families (Roy et al., 2014), we could not find studies that tested the effect of SES on verbal learning among children in this age group (but see income-dependent performance in adults in Al Hazzouri et al., 2017). Explicit vocabulary interventions suggest that children from high SES families gain substantially more than children from low SES families, consequently widening the gap (Hart and Risley, 2003; Marulis and Neuman, 2010; Mcgillion et al., 2017). We were also unable to find studies assessing the effect of parental education on verbal list learning, although one study did test learning of words from presented pictures among schoolchildren aged 8–12, revealing that children of better educated parents outperformed children of less educated parents (van Tetering et al., 2018). It is of interest to find out whether similar findings would arise for list learning in children from low-SES families, and furthermore, whether additional conditions such as low ME or reading and spelling difficulties would affect learning.

### Children at Risk of Reading and Spelling Difficulties

Typical learners acquire the basic mechanics of reading and spelling in their first 2 years of school (Carver, 1990; Overvelde and Hulstijn, 2011). Difficulties in the acquisition of reading and spelling are primarily attributed to deficits in the intentional acquisition of knowledge, such as that required in verbal learning (Kramer et al., 2000; West et al., 2018), but also to deficits in the acquisition of implicit knowledge through practice (Needle et al., 2006; Hedenius et al., 2013). These two mechanisms may interact such that implicit knowledge (e.g., in phonology) may underlie intentional operations (e.g., intentional manipulation of individual phonemes), whereby impairments were detected for implicit phonological representations in children with reading difficulties (Peterson and Pennington, 2015). In addition to the well-studied effect of phonological knowledge on literacy skills (Peterson and Pennington, 2015), performance levels of grapho-motor skills and vocabulary are also considered predictors of growth in literacy during the early school years (Hamstra-Bletz and Blöte, 1993; Parush et al., 2010; Oyler et al., 2012). Moreover, performance levels in grapho-motor skills (Costa et al., 2018) and verbal memory (Scarborough, 1998) domains are commonly impaired in children with reading and spelling difficulties.

During the second grade, up to 30% of low achievers steadily improve their reading in the direction of average reading scores (Spira et al., 2005), thus suggesting that an evaluation of reading and spelling difficulties is premature for many of these children. This supports the view that focuses on low achievers as children *at risk* of reading and spelling difficulties. In the current study, we will focus on this population.

### Motor Skill Learning and Verbal Knowledge Acquisition in Children With Reading and Spelling Difficulties and in Children With Reading and Spelling Difficulties From Low-Socioeconomic Status Families

Motor difficulties are common among children with reading and spelling difficulties, with a third or more of the children showing reading and spelling difficulties being diagnosed with motor difficulties (for a recent review, see Downing and Caravolas, 2020). Studies on motor skill learning among children with reading and spelling difficulties focus almost exclusively on the initial training session, implying intact performance in longer training sessions (for a recent review, see He and Tong, 2017). Few studies extend research beyond the initial training session, suggesting that later phases, particularly the consolidation phase, may be delayed or even impaired (Vicari et al., 2005; Hedenius et al., 2013). Notably, similar findings have been reported for children with developmental language disorder (Hedenius et al., 2011; Lum et al., 2012), including participants as young as 5 years of age (Adi-Japha et al., 2011b; Adi-Japha and Abu-Asba, 2014), as well as in those with attention deficits (Adi-Japha et al., 2011a; Fox et al., 2016).

Kramer et al. (2000), who studied the performance of 9-year-old children with reading disabilities on the California Verbal Learning Test – Children’s Version found that children with reading disabilities had impairments in their ability to learn new information but were able to retrieve information with typical proficiency and retain it over time. van Strien (1999) found that 10-year-old boys with reading disabilities recalled and recognized fewer words on the Rey Auditory Verbal Learning Test (RAVLT) than did controls. Oyler et al. (2012) used the Bergen-Tucson Verbal Learning Test (BTVLT) to compare middle-class adolescent students with reading disabilities to typical learners. The reading-disability group learned significantly fewer list items and did so more slowly than did the control group. It should be noted that verbal list learning has not always been found to be weak in children with reading difficulties (Brady, 1991). Furthermore, Tijms (2004) noted that effects found reflect an inaccurate encoding of the phonological characteristics of verbal information rather than a verbal memory deficit. In line with this notion, a recent large-scale study indicated that verbal short-term/working memory played a comparatively minor role in explaining diagnostic criteria for dyslexia (Landerl et al., 2013).

Although previous literature suggests that reading and spelling difficulties and SES are independently associated with variations in motor skills and vocabulary learning, their joint influence has been studied less. We were not able to find studies that tested the effect of SES (or ME) on motor learning in children with reading and spelling difficulties. However, we did find a few studies that tested the effect of SES and reading difficulty level on outcomes of reading intervention programs. These studies reported independent effects of reading difficulty and SES, albeit in different directions: While one study reported lower gains for lower SES and for lower reading skills (Hatcher et al., 2006), another study reported the opposite – with children who have lower reading skills and come from a low-SES background gaining the most (Romeo et al., 2018). This latter result is, however, at odds with most of the literature that reports lower intervention gains in lower SES (Torgesen and Mathes, 1999; or no effect of SES, Morris et al., 2012) and in children with lower reading abilities (Vellutino et al., 2007; Compton et al., 2012). It has been suggested that environmental factors, such as home literacy, access to reading material, and school quality may be responsible for systematic heterogeneity in the root cause of reading difficulties across children from varying SES (Peterson and Pennington, 2015; Ursache and Noble, 2016); and therefore, children with reading and spelling difficulties of varying SES may learn and retain new information differently (Friend et al., 2008; Mascheretti et al., 2013).

### The Current Study

The current study focuses on learning a new grapho-motor pattern and on verbal learning among second graders of low-SES background with lower (up to 12 years of schooling, the mandatory education level in Israel) or higher ME. Differences in relation to this education level have been found for motor skills (Valadi and Gabbard, 2020) and vocabulary (Andonova, 2015; Cadime et al., 2018; and see also maternal talk to children, Korat, 2009). Within the above population, the current study differentiates between children with and without reading and spelling difficulties.

Our research hypotheses are:

(a) For grapho-motor learning,

(1) Lower ME would moderate overall performance level. Regarding training gains and their retention: it is not clear what the effect of ME would be.

Children with lower ME were shown to have lower motor skills (e.g., Valadi and Gabbard, 2020). It has been suggested that these children have less play options at home for developing their fine motor skills and have lower access to physical activity, for example, due to lack of community playgrounds (McNeill et al., 2006). Regarding training gains and their retention, the effect of lower ME is not clear due to the mixed results reported in the literature (De Bourdeaudhuij et al., 2011; Birnbaum et al., 2017), even within the same study (Bardid et al., 2013). These mixed findings may stem from differing difficulty levels of the motor tasks in relation to the pre-training level of the child, as well as from more general socio-cultural background factors (McNeill et al., 2006; Bardid et al., 2013).

(2) Reading and spelling difficulties would moderate overall performance level. We do not expect effects of reading and spelling difficulties on training gains; however, lower performance levels 24-h post initial training is expected.

Children with reading and spelling difficulties were shown to have motor difficulties. Several hypotheses were made regarding the source of comorbidity between reading and spelling difficulty and lower motor skills, all relate to the multifactorial nature of development with complex interactions within the environmental and the biological/genetic substrate and between these substrates (Downing and Caravolas, 2020). The effect of reading and spelling difficulties on training gains was shown to depend on training length (He and Tong, 2017). Given the very extensive course of training suggested below, we expect no effect of reading and spelling difficulties on training gains. However, previous studies indicate poorer retention 24-h post-training (Vicari et al., 2005; Hedenius et al., 2013), possibly due to atypical consolidation processes (Hedenius et al., 2013) and we expect similar findings here.

(b) For verbal learning,

(1) Lower ME would moderate overall performance level. Due to a lack of studies, the effect of ME on practice and retention is not clear.

Beginning in infancy, children with lower ME are less exposed to verbal input by their parents and were shown to have lower verbal skills (Roy et al., 2014; Valadi and Gabbard, 2020), therefore lower ME is expected to moderate overall task performance. Regarding practice gains and delayed recall, the effect of lower ME is not clear due to mixed findings. Lower ME was associated with less effective learning of verbal materials in intervention studies (Marulis and Neuman, 2010; Mcgillion et al., 2017). However, van Tetering et al. (2018), who studied a pictorial verbal learning task, reported an association between parental education level and task performance throughout the task, suggesting no education level effects on learning per-se. While most studies report lower gains in lower SES (or ME) level, or no difference in gains for differing SES (or ME) levels, few report the opposite (Romeo et al., 2018). These mixed findings may be related to the pre-training level of the children.

(2) The effect of reading and spelling difficulties on the overall performance, and on practice gains and their retention is not clear.

Verbal difficulties are characteristic of children with reading and spelling difficulties (Downing and Caravolas, 2020), however verbal learning relate more to memory for words than to other verbal skills (e.g., phonology). In this context, only weak association was reported between reading difficulties and verbal short-term/working memory (Landerl et al., 2013). Furthermore, the literature presents mixed findings regarding the effect of reading and spelling difficulties on verbal learning (Brady, 1991; Kramer et al., 2000; Oyler et al., 2012), and when found, these deficits were attributed to inaccurate phonological encoding rather than to a memory deficit (Tijms. 2004). Therefore, no clear hypothesis can be made regarding the performance of children with reading and spelling difficulties on the RAVLT.

The joint influence of ME level and reading and spelling difficulties on motor and verbal skills and their learning has not been studied yet. We were not able to find previous studies regarding motor skills or their learning, while for verbal learning the literature suggests mixed findings (Hatcher et al., 2006; Romeo et al., 2018).

For the purpose of the current study, all second graders who attended two low-SES schools (one for boys and one for girls) were assessed using the invented letter task (ILT, Julius and Adi-Japha, 2015) and the RAVLT.

In the ILT, participants connect three dots in a given sequence to form a “letter” pattern. The task used here involved two successive days of training and retention evaluation, as well as transfer to a novel task condition 2-weeks post-training. Performance of the ILT was shown to correlate with handwriting speed and accuracy in kindergarteners and second graders, as well as with reading speed in the second grade, and to predict handwriting and reading proficiency in the year following the ILT practice (Julius et al., 2016). In comparison to typically developing children, the ILT showed an atypical acquisition pattern in children with developmental language disorder (DLD; Adi-Japha et al., 2011b; Adi-Japha and Abu-Asba, 2014), commonly associated with deficits in motor performance and skill learning (Krishnan et al., 2017). Moreover, ILT studies showed different abilities as well as transfer gains in children with developmental coordination disorder (DCD; Adi-Japha and Brestel, 2020), commonly associated with difficulties in mastering handwriting (Farmer et al., 2016). It should be noted that due to the high comorbidity of reading and spelling difficulties and DCD (Downing and Caravolas, 2020), the research questions of the current study may be also relevant to children with DCD.

The RAVLT (Rey, 1964) is a standardized word-list multiple-trials test, frequently used in neuropsychological batteries. In the RAVLT, children are read a list of words five times and are asked to recall these words at a lag, allowing the assessment of learning and retention. The test has been previously used in this age-group in relation to literacy skills (van Strien, 1999). Several list-learning tests exist, but only the RAVLT was standardized in Hebrew.

Assessment studies have shown that difficulty in word reading may affect spelling until the word-reading and/or spelling problems are remediated, with spelling as the more persistent problem (Berninger and Richards, 2010). Therefore, word reading (speed and accuracy scores) and spelling subsets of a normed literacy screening test used to detect reading and spelling difficulties.

## Materials and Methods

### Participants

A sample of 151 second graders (age *M* = 8.14; SD = 0.45) from two primary schools in the same city (a low-SES city, according to the national rankings in Israel) was recruited for the study. The schools’ SES were ranked 8 and 9 (10 is considered the lowest). The schools participate in a national nutrition program that provides all students with subsidized lunches. Boys (*n* = 107) and girls (*n* = 44) learn separately. The study was conducted in 2016/17. All monolingual Hebrew-speaking second graders in these schools were invited to participate. One boy (bilingual), one girl who had a cochlear implant, and thirteen boys and two girls diagnosed with ADHD and were being medically treated were not included in the data analyses. For two children, the parents did not provide maternal years of education data. The final sample comprised 134 children (40 girls).

Participants’ parents completed a SES information questionnaire in which they reported their occupation and education level, among other SES factors. Maternal education was defined in terms of years of study. Twelve years of maternal education or less (basic, non-vocational compulsory education level as of 2007) were coded as low ME (*n* = 69). Participants were defined as having reading and spelling difficulties on the basis of low scores on the Ma’akav test (a normed test for the assessment of basic literacy skills). Children with a standardized score lower than 25% on at least one measure of the word-reading test (speed or accuracy), combined with a standardized score lower than 25% on the spelling-to-dictation test were defined as children with reading and spelling difficulties (*n* = 39). These included 20 children with low ME and 19 with higher ME. Of the 95 typical learners, 49 children had mothers with low education levels and 46 had mothers with higher education.

Of these 134 children, 129 children completed all phases of the ILT (93 typical learners, of whom 49 were low ME; and 36 children with reading and spelling difficulties, of whom 18 were low ME). The 129 children also completed all phases of the RAVLT (93 typical learners, of whom 50 were low ME; and 36 children with reading and spelling difficulties, of whom 17 were low ME).

Sample size was calculated on the basis of several studies, all investigating students with and without learning difficulties (Swanson and Harris, 2013). With a power of 0.80 and a two-tailed α = 0.05, a sample size of 128 participants in a 2 × 2 design was found large enough to detect medium effects (*f* = 0.25, Cohen, 1988) or larger. This calculation was based on an *a-priori* power analysis using G^∗^Power 3.1 (Faul et al., 2009). The children were tested for nonverbal IQ as measured by the Colored Progressive Matrices Nonverbal IQ Test (Raven et al., 2008) and Visual Motor Integration (VMI, Beery and Beery, 2010). Descriptions of the children’s SES backgrounds and learning abilities are presented in Table 1.

**TABLE 1:** Means, standard deviations, and analysis of variance (ANOVA) of descriptive data.

Measures	Typical learner				Reading and spelling difficulties				ME	Reading and spelling difficulties	ME* reading and spelling difficulties
	LME		HME		LME		HME				
	*M*	*SD*	*M*	*SD*	*M*	*SD*	*M*	*SD*	F *(η_*p*_^2^*)	F (η_*p*_^2^)	F (η_*p*_^2^)
Age (years)	8.19	0.49	8.04	0.45	8.21	0.26	8.14	0.42	1.7 (0.01)	0.52 (0.00)	0.26 (0.00)
Mean Z SES	–0.18	0.45	0.28	0.64	–0.31	0.48	0.19	0.40	23.40*** (0.15)	1.13 (0.01)	0.04 (0.00)
Non-verbal reasoning	102.19	11.40	106.43	10.78	101.25	14.78	102.53	10.98	1.52 (0.01)	1.17 (0.01)	0.44 (0.00)
Visual motor integration	91.92	8.85	93.57	11.14	88.15	11.62	88.94	9.39	0.39 (0.00)	4.59* (0.03)	0.05 (0.00)
Vocabulary	8.23	2.79	8.73	2.79	7.55	2.69	8.28	2.27	1.39 (0.01)	1.18 (0.01)	0.05 (0.00)
Verbal STM	10.31	3.10	12.11	3.31	9.26	3.49	10.32	3.18	5.27* (0.04)	5.19* (0.04)	0.36 (0.00)
Literacy test raw score	54.98	15.53	59.13	16.22	28.79	15.51	31.83	13.23	1.43 (0.01)	78.89*** (0.39)	0.03 (0.00)
Literacy test percentile	28.23	18.84	33.22	22.16	13.06	9.87	11.67	4.85	0.26 (0.00)	27.12*** (0.18)	0.82 (0.01)
Spelling (# errors)	6.41	3.61	6.11	4.09	12.37	3.47	12.94	3.70	0.58 (0.01)	36.1*** (0.22)	0.25 (0.00)
Reading Comprehension	30.47	10.60	32.58	9.21	20.21	8.29	24.94	11.67	3.1 (0.02)	21.27*** (0.14)	0.46 (0.00)

LME, Low Maternal Education (=12 and under); HME, High Maternal Education (above 12); η_p_^2^, Partial Eta Squared; Non-Verbal Reasoning, Colored Progressive Matrices (Raven standardized score; M = 100, SD = 15); SES, Mean Z SES, Averaged score of all 7 SES measures; VMI, Visual Motor Integration (Beery) Standardized score (M = 100, SD = 15); Vocabulary Number of correctly described words of the WISC-R 95 subtest standardized score (M = 10, SD = 3); Verbal STM, Verbal short-term memory – the Number Recall subtest of the WISC-R 95 standardized score (M = 10, SD = 3); Literacy Test, Meitzav Literacy national test (reading comprehension, spelling and writing composition). Percentile, percentile score; Spelling (# errors), Number of errors on the spelling subtest of the Meitzav Literacy national test; Reading Comprehension, Raw scores on the reading comprehension subtest of the Meitzav Literacy national test ***p < 0.001, *p < 0.05.

### Testing Procedures

The study was approved by the Israel Ministry of Education (7883/054 10.32) and parents signed the Ministry of Education consent forms. Child assessments were conducted at the schools, in one quiet room, by an experienced occupational therapist (Author 1) in four or five individual 20–50-min pull-out sessions during school hours. Blind coders (healthcare graduates) evaluated performance and coded the data.

#### Maternal Education

Data on maternal education level, known to be an important factor in predicting development in early childhood and academic success, was taken from the Hollingshead (1975) SES questionnaire (see below), and was coded as high if above upper secondary (high school level), and otherwise as low.

The discussion on the effects of SES in the context of child outcomes commonly refers to education level, rather than to a more accumulative measure such as parental schooling years. Within schooling levels, the distinction between below- or at-high-school level and above-high-school level refers to both qualitative and quantitative changes (Duncan et al., 2012). A low ME threshold has been taken here at high-school level (upper secondary level of education without any vocational or tertiary education) as this level of education sets the benchmark for comparison among groups in relation to SES outcomes in education (OECD, 2020). In Israel, where the current study took place, high-school level education has been mandatory as of 2007.

#### The Socioeconomic Status Questionnaire

The SES questionnaire is a seven-factor index based on Hollingshead (1975), adapted for Israeli culture by Korat et al. (2007) (α = 0.90), and includes the education levels of the parents, their occupations and/or professional qualifications, number of children, rooms, and family income level. This measure was used to test if low/high ME groups differed in their overall SES scores. The professional qualification and current occupation scale ranges from 1 (unskilled workers and menial laborers) to 5 (upper-level executives and professionals). Parents were asked to rank their combined income level relative to the official Israeli average at the time of testing, on a scale from 1 (*much below the national average*) to 5 (*much above the national average*). To calculate a SES value, the data were transformed to a 5-point scale (from 1 = *low* to 5 = *high*). The 7 SES indices Z-scores were averaged (see Table 1) to adjust for differences in the scales and distributions across variables (Korat et al., 2007).

#### Non-Verbal Reasoning – Raven’s Educational Colored Progressive Matrices

Raven’s Non-Verbal Reasoning test (Raven et al., 2008) is commonly used when studying developmental disabilities and in neuropsychological assessments to characterize study populations and evaluate potential differences between study populations. In particular, the Raven’s test has been used in ILT studies (Adi-Japha and Abu-Asba, 2014; Adi-Japha and Brestel, 2020) and in studies that assessed the RAVLT (e.g., Chiaravalloti et al., 2020). In the current study, the Raven was used to obtain a nonverbal assessment of intelligence, in a version suited for children aged 5–11. It comprises 36 items in three sets (A, AB, and B) of 12 colored large-print drawings each. Each item is presented with an incomplete design and six alternatives, from which the alternative that best completes the design must be chosen. The items entail escalating skills in encoding and analyzing information. The sum of scores on the full test (score range 0–36) was converted into Z scores. Internal consistency in 8- to 9-year-olds was high (0.88), and split-half reliability was 0.9 (Cotton et al., 2005).

#### Beery Visual Motor Integration

The Beery-Buktenica developmental test of VMI (Beery and Beery, 2010, 6th edition) was used. The VMI, a standardized test that evaluates the VMI skills of individuals aged two to adulthood, includes 30 geometric forms that are to be copied. The forms are set in a progressively difficult sequence. In the current study, the test was stopped after a participant failed to correctly copy three consecutive shapes. Reliability was found to be 0.80–0.92; mean score (*M*) = 100; and standard deviation (*SD*) = 15. Children with reading and spelling difficulties often encounter difficulties in handwriting production (Cheng-Lai et al., 2013; Martínez-García et al., 2020). Because the ILT is a writing-like visual-motor task the VMI is frequently used in ILT studies, and it may serve as a covariate in the analysis of ILT performance in case of potential group differences in visual-motor skills (Adi-Japha et al., 2011b; Adi-Japha and Abu-Asba, 2014).

#### The Ma’akav Test for Basic Reading and Spelling Skills

Ma’akav (Shany et al., 2003), a normed referenced evaluation for second to sixth grades, is recommended as part of the curriculum in primary schools in Israel. It comprises five subtests, of which only two were used in the current study: single word reading – 38 words (α Cronbach = 0.91) and a spelling-to-dictation text of 27 words assessed for spelling (reliability of alternate forms for the accuracy measure = 0.59).

Spelling in Hebrew may be much more challenging than reading because the vast majority of Hebrew words can be spelled in more than one way (Share and Levin, 1999), implying that reading and spelling should be studied together. Therefore, two subtests of Ma’akav screening test were used to detect reading and spelling difficulties: (a) a word-reading test composed of individual words with voweling and (b) spelling-to-dictation of a short text. Following the screening instructions, children were defined as low achievers if they were among the lowest 25% of achievers in speed or accuracy of word-reading and in the lowest 25% of achievers on the spelling test (see also Shany and Share, 2011; Blicher et al., 2017).

#### The Invented Letter Task

**FIGURE 1:**
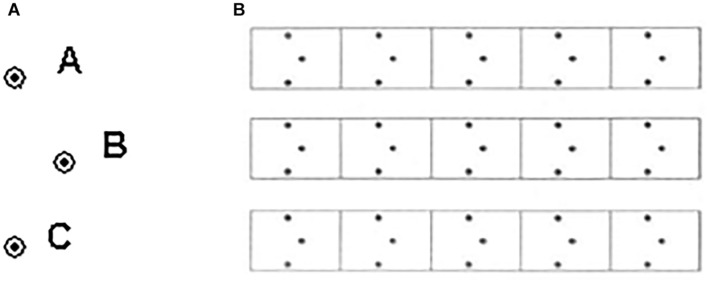
**(A)** ILT, Invented Letter Task Stimuli. **(B)** ILT Block.

**FIGURE 2:**
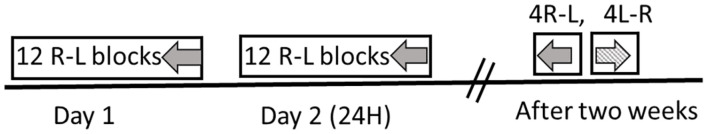
ILT research plan. On Day 1, Day 2, and the first four blocks, 2 weeks after training (retention testing): movement progress is from right to left (R–L; as in Hebrew writing). On the four blocks of the transfer task, movement progress is from left to right (L–R).

The ILT was used to study the time-dependent course of motor skill acquisition. The task consists of point-to-point planar movements (Figure 1A: A→B→C, segment length 1.2 cm, circle outer diameter 3 mm, shape width 6 mm) to form an invented letter. Movement progress is from right to left (as in Hebrew spelling). Each ILT block contains 15 repeats of the same pattern (Figure 1B). Overall, 28 blocks of the task were performed: 12 on the first day of training; twelve after a 24-h lag, i.e., on the second day of training (also referred to as the *consolidation* phase); and four blocks, 2 weeks after the first day of training (the *retention* phase). Transfer was tested (*transfer* condition, Figure 2) immediately after the retention testing and consisted of performing the task from left to right on four additional blocks.

The participants practiced the task at the beginning of each day for one block until they completed one line with no errors. They were instructed to perform the task as quickly and accurately as possible, going through all three encircled dots in one continuous line (Adi-Japha et al., 2011b; Julius and Adi-Japha, 2015; Julius et al., 2016; Adi-Japha and Brestel, 2020). On all testing days, the blocks were separated by a minimum of 15 s and up to a 30-s break. After each practice session was completed, the experimenter placed an identical sheet of paper on the digitizer in front of the participant for completion. The same task was repeated for the practice sessions during the three intervals. No feedback was provided on any performance measure other than general encouragement (“You’re doing fine”; “Pay attention to the task”; “Remember to be as quick and accurate as possible!”). The children completed the practice sessions within 20 min. Practice in a connecting dot pattern is standard in learning letter-writing in many school curricula (e.g., *Dizzy from A to Z*, Tsur, 2006), Hebrew (the language used by the participants; e.g., *Magic and Friends*, Center for Educational Technology, 2019), and Arabic (e.g., *Arabic is our Language*, Center for Educational Technology, 2010).

Children performed the task on a half-A4 piece of paper placed vertically on a Wacom tablet (sampling rate 200 Hz, nominal accuracy 0.02 mm). An ink stylus resembling a ballpoint pen, which leaves a visible ink trace on the page (see Supplementary Figures 1, 2), was used. The tablet was placed on a slanted ergonomic board that was set on a table adjusted to the child’s seat height in order to achieve an ergonomically optimal spelling position (a 20-degree slant). Participants’ feet were placed on a stable platform (at a 90-degree angle). The writing product was evaluated using a computer program that computed the average value per block of the writing accuracy and overall time (see Supplementary Figure 3).

##### Invented letter task coding

The ILT was coded for accuracy and performance times. We used two accuracy measures. (1) Surplus segments. The digitizing tablet provided a flag measure of the on-page or off-page contact that was calibrated by the axial pressure of the writing stylus on the tablet surface and was used to evaluate the number of segments children produced. Each block consisted of 15 shapes and should include 15 segments. Surplus segments were coded as the number of segments above 15 used per block (Julius and Adi-Japha, 2015; Adi-Japha and Brestel, 2020). (2) Erroneous shapes. Shapes *not* produced in one continuous movement or shapes that are too narrow or too wide relative to the encircled point B, the midpoint of the shape in Figure 1A (this corresponds to not going through the encircled point; Julius and Adi-Japha, 2015). While the use of surplus segments is associated with basic production difficulties (e.g., in children with DCD; Adi-Japha and Brestel, 2020), and was uniquely related to retention deficits in typically developing kindergarten children (Julius and Adi-Japha, 2015), overall accuracy is taken more as a measure of movement planning using spatial guidelines (Adi-Japha and Brestel, 2020). In addition to accuracy, we evaluated performance times. Overall time was computed based on the digitized data, from the first touch of the pen tip on the page until block completion.

The ILT analysis consisted of five time-points (TP): TP1 (initial training, the mean of the first four blocks of Day 1); TP2 (the mean of the last four blocks of Day 1 training); TP3 (24-h consolidation, the mean of the first four blocks of Day 2); TP4 (the mean of the last four blocks of Day 2 training); TP5 (2-week retention, the mean of four blocks, 2 weeks after training). Transfer was evaluated at the session held 2 weeks after training (the mean of four blocks).

For each time-point of the three study measures, the split-half reliability was calculated. For surplus segments, the split-half reliabilities for the 5 time-points and transfer task were: 0.67, −0.79, and 0.91, respectively; for erroneous shapes, 0.67, −0.80, and 0.79, respectively; and for performance times, 78, −0.84, and 0.81, respectively. Reliability increased with practice.

#### Rey Auditory Verbal Learning Test

Rey Auditory Verbal Learning Test (Rey, 1964) is a normed word-list multiple-trials test for ages 8–91 for assessing verbal (declarative-intentional) memory skills. The Hebrew version of the RAVLT was used. Raw scores were translated to Z scores based on age and gender (Vakil et al., 2010). Concurrent and delayed measures were used. The test consists of 15 common nouns that are read to participants at the rate of one word per second in five consecutive trials (Trials 1–5). Each reading is followed by a free recall task. Twenty minutes later, participants are asked to recall the first list without an additional reading (Recall Trial). Standard Z scores were calculated in accordance with Israeli gender norms as described by Lezak et al. (2004) for the following developmentally sensitive measures: *Learning.* Learning was measured in terms of cumulative recall after the fifth reading vs. the first reading (Trial 5 vs. Trial 1); *Retention*. Retention of learned material over time (Recall Trial vs. Trial 5). In a study of 225 6- to 12-year-olds, test-retest reliability of Trials 1–5 was 0.70, and of the delayed recall 0.62 (van den Burg and Kingma, 1999).

#### Vocabulary

Subtest 7 from the Hebrew version of the Wechsler Intelligence Scale for Children (WISC R-95; Wechsler, 1998) standardized for age 6–13. This is a progressively difficult test in which participants describe or explain the meaning of 25 words read to them. The maximum score for this subtest is 50 points. Reliability for this test was found to be *r* = 0.85 and construct validity **´α** = 0.62. Standard scores were used with *M* = 10, *SD* = 3.

#### Digit Span Number-Recall (Sequential Verbal Short-Term Memory) Test

Subtest 11 from the WISC R-95 Hebrew version (Wechsler, 1998) adapted for ages 6–13, found to be a particularly strong predictor of language and academic achievements, and considered as a measure of phonological short-term memory (STM; Alloway and Alloway, 2010), was used. In the first subtest task, participants were presented with two spoken series of three to nine digits and were asked to repeat them. In the second subtest, the Backward Digit Span, participants were asked to repeat two series of two to eight digits in reverse. Each of the 14 trials was coded as *recalled correctly* or *incorrectly*, and the total number of digits across all correct trials was recorded. The total of the two subtests was coded; reliability was found to be *r* = 0.85 and construct validity **´α** = 0.62. Standard scores were used with *M* = 10, *SD* = 3.

Because the RAVLT is a verbal word-learning task, vocabulary as well as verbal short-term memory measures are often correlated with performance level on this task. As this task is here used to assess group differences in verbal learning and memory, these two variables were set as covariates (Keenan et al., 1996; Krueger and Salthouse, 2010; Vakil et al., 2012).

#### Literacy Test-Meitzav, a National Academic Reading and Spelling Test

The 2012 Meitzav test for Grade 2, Part A, was used to evaluate literacy skills. Meitzav is part of a national testing program conducted by the National Authority for Measurement and Evaluation in Education, in which primary, junior high, and senior high schools participate. Until 2016, second graders were part of the testing system, with testing conducted during the last trimester of the school year. Since then, these children have been tested internally at the beginning of the year, using a different version of the test. The 2012 Meitzav was comprised of six sections (dictation accuracy, reading and writing accuracy, reading comprehension, written expression, and linguistic knowledge). The Meitzav total score of part one is normalized. An additional scale according to SES were calculated nationally (*M* = 67, *SD* = 22 *for the lowest SES rank).* The Reading comprehension subtest scores under 65 were considered the 25th percentile, scores and under 49 were the 10th percentile and were reported in this study (RAMA, 2016).

## Results

We provide sample descriptive statistics (Table 1) and confirm that low SES children show lower motor and verbal learning scores than that of the general population. We then move to the main study analysis: Practice dependent learning on the motor and verbal tasks (see Supplementary Table 1 for Means and Standard Deviations of Practice dependent learning on motor and verbal tasks Measures).

### Background Variables

A series of Kolmogorov-Smirnov tests indicated that the main study measures (overall ILT scores of surplus segments, erroneous shapes, and performance times, and RAVLT recall scores) did not violate assumptions of normality. A chi-square test for association was conducted between ME and reading and spelling difficulties. All expected cell frequencies were greater than five. It is important to note that reading and spelling difficulties groups did not differ in their ME level of education *χ*^2^(1) = 17, *p* = 0.681. No statistically significant differences in ratios between boys and girls were found in all groups.

Table 1 presents descriptive statistics of the sample and the results of a 2-way ANOVA with reading/spelling difficulties (reading and spelling difficulties, present/absent) and ME (low/high). As can be seen, children with lower ME had significantly lower overall SES scores. Children with lower ME [*F*_(1, 129)_ = 5.27, *p* = 0.023, η_*p*_^2^ = 0.039] as well as children with reading and spelling difficulties [*F*_(1, 129)_ = 5.19 *p* = 0.024, η_*p*_^2^ = 0.039] had lower number-recall scores (verbal STM). Children with reading and spelling difficulties additionally had lower visual-motor skills [*F*_(1, 129)_ = 4.59, *p* < 0.001, η_*p*_^2^ = 0.034] and generally, as expected, lower literacy skills [*F*_(1, 129)_ = 78.89, *p* < 0.001, η_*p*_^2^ = 0.39] {spelling, [*F*_(1, 129)_ = 36.10, *p* < 0.001, η_*p*_^2^ = 0.22] and reading comprehension [*F*_(1, 129)_ = 21.27, *p* < 0.001, η_*p*_^2^ = 0.14]} than typical-learner peers.

### Comparison to Normative Values in the General Population

On all measures reported in Table 1, the group of typical learners with higher maternal education achieved the highest scores. Nevertheless, compared with the standard means among the general population, even participants in this group scored significantly *lower* in motor and verbal testing: on the VMI test [*t*_(45)_ = 3.92, *p* < 0.001], and the vocabulary subtest of the WISC-R 95 [*t*_(44)_ = 3.04, *p* < 0.001], as one would expect among low-SES samples (Noble et al., 2006; Dinehart and Manfra, 2013; Roy et al., 2014; Julius et al., 2016; Manfra et al., 2017).

### Motor-Learning: The Invented Letter Task Accuracy and Time Measures

Accuracy and time on the motor-learning task were analyzed using a mixed-design ANOVA, with time-point as the between-subject variable and ME and reading and spelling difficulties as between-subject variables, using a 5 (time-points: beginning of Day 1, end of Day 1, beginning of Day 2, end of Day 2, 2 weeks post-training retention) × 2 (ME = low/high) × 2 (reading and spelling difficulties = with/without) design; Transfer task was studied using a 2 (time-point = ILT retention, ILT Transfer) × (ME = low/high) × 2 (reading and spelling difficulties = with/without) mixed-design ANOVA whereby ILT retention and transfer were assessed 2-weeks post initial training. The Greenhouse-Geisser correction was used when the assumption of sphericity was violated.

#### Accuracy Performance

##### Surplus segments

**FIGURE 3:**
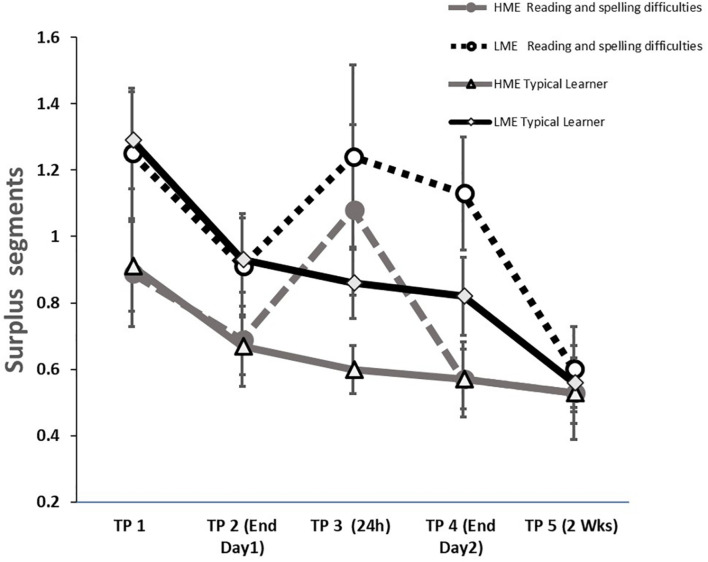
ILT number of surplus segments (assessed as surplus hand raises, mean and standard deviation) among the four groups: *HME typical learner*, typical learner with high maternal education; *LME* typical learner, typical learner with low maternal education; *HME reading and spelling difficulties*, reading and spelling difficulties with high maternal education; *LME reading and spelling difficulties*, reading and spelling difficulties with low maternal education. The figure depicts the five points in time: *TPI (Init Day 1*) Initial Day 1: *TP2 (End Day 1)* end of Day 1, *TP3 (24 h) post-training* initial of Day 2, *TP4 (End Day 2)* end of Day 2; *TP5 (2 Weeks)* and 2 weeks post-training retention testing.

Young children often fail to produce the ILT shape in one segment (Julius and Adi-Japha, 2015). Analysis of the surplus segments (Figure 3 and Table 2) indicated a main effect of ME [*F*_(1, 125)_ = 6.08, *p* = 0.015, η_*p*_^2^ = 0.05]. Accordingly, children with higher maternal education were overall more accurate (i.e., fewer surplus segments). Additionally, an overall effect of time-point [*F*_(4,500)_ = 10.45, *p* < 0.001, η_*p*_^2^ = 0.08] and a trend toward a Time-point × Reading and spelling difficulties group interaction [*F*_(4,500)_ = 2.39, *p* = 0.050, η_*p*_^2^ = 0.02] emerged.

**TABLE 2:** Analyses of ILT surplus segments.

	ILT learning								Transfer task							
	Surplus Segments				With VMI as a covariate				Surplus Segments				With VMI as a covariate			
	DF	*F*	*p*	η^2^	DF	*F*	*p*	η^2^	DF	*F*	*p*	η^2^	DF	*F*	*p*	η^2^
Time-Point	4, 500	10.45	0.000	0.08	4, 496	0.46	0.766	0.00	1, 125	5.99	0.016	0.05	1, 124	0.01	0.919	0.00
Time-Point × VMI					4, 496	0.41	0.801	0.00					1, 124	0.06	0.808	0.00
Time-Point × ME	4, 500	1.22	0.300	0.01	4, 496	1.26	0.284	0.01	1, 125	4.01	0.047	0.03	1, 124	4.02	0.047	0.03
Time-Point × reading and spelling difficulties	4, 500	2.39	0.050	0.02	4, 496	2.40	0.049	0.02	1, 125	0.65	0.420	0.01	1, 124	0.69	0.407	0.01
Time-Point × ME × reading and spelling difficulties	4, 500	0.28	0.893	0.00	4, 496	0.27	0.899	0.00	1, 125	0.41	0.521	0.00	1, 124	0.39	0.531	0.00
VMI					1, 124	0.13	0.718	0.00					1, 124	1.87	0.173	0.01
ME	1, 125	6.08	0.015	0.05	1, 124	6.43	0.012	0.05	1, 125	4.22	0.042	0.03	1, 124	2.64	0.107	0.02
Reading and spelling difficulties	1, 125	0.79	0.376	0.01	1, 124	0.68	0.410	0.01	1, 125	0.65	0.420	0.01	1, 124	0.05	0.831	0.00
ME × reading and spelling difficulties	1, 125	0.04	0.851	0.00	1, 124	0.03	0.865	0.00	1, 125	0.41	0.521	0.00	1, 124	0.03	0.864	0.00

*VMI, Visual Motor Integration raw score; ME, Maternal Education level (low/high); reading and spelling difficulties, Reading and Spelling Difficulties.*

To verify that differences between groups did not occur due to the lower visual-motor skills of this group (Downing and Caravolas, 2020; and here as indicated in Table 1), the analysis was repeated with VMI scores as a covariate. The analysis indicated a main effect of ME [*F*_(1, 124)_ = 6.43, *p* = 0.012, η_*p*_^2^ = 0.05] and a Time-point × Reading and spelling difficulties Group interaction [*F*_(4,496)_ = 2.40, *p* = 0.049, η_*p*_^2^ = 0.02] emerged. These data suggest that although groups differed in their VMI scores, this did not affect the difference between lower and higher ME in overall performance or the Time-point × Reading and spelling difficulties Group interaction.

To follow the Time-point × Reading and spelling difficulties Group interaction found (with the VMI held as a covariate) group differences were evaluated at each of the time-points. The analysis indicated a significant group difference only at the beginning of Day 2 (group difference at the beginning of Day 2: *p* = 0.009; This significance level is in-line with the Bonferroni correction at *p* < 0.01 level for group comparison at 5 time-points). This group difference emerged due to an increase in surplus segments of the reading and spelling difficulties group, *t*(35) = 2.28, *p* = 0.029. No group difference emerged at any of the other time-points (*ps* > 0.1).

Due to the relatively discrete low values of the surplus segment variable, at each time-point a-parametric Mann-Whitney analysis was undertaken. The analyses indicated similar findings to those reported for the parametric analyses, with a significant group difference only at the beginning of Day 2 (Z = 1.99, *p* = 0.047), but not at the other time-points (*Z* < 0.1.65, *p*’s > 0.1).

The surplus segments transfer analysis indicated an increase in the number of surplus segments [time-point main effect: *F*_(__1__,125)_ = 5.99, *p* = 0.016, η_*p*_^2^ = 0.05] and especially for the lower ME children, as indicated by a Time-point × ME Group interaction [*F*_(1,125)_ = 4.20, *p* = 0.042, η_*p*_^2^ = 0.03]. When VMI scores were used as a covariate, the analysis indicated only the Time-point × ME Group interaction [*F*_(1,__124__)_ = 4.22, *p* = 0.042, η_*p*_^2^ = 0.03]. This interaction emerged because no ME-dependent group difference emerged at ILT testing 2 weeks post-training [*t*_(12__6__)_ = 0.79, *p* = 0.426]; however, at the transfer condition, a significant difference emerged [*t*_(12__6__)_ = 2.51, *p* = 0.013]. Thus, the group difference at the transfer condition resembles the groups difference found at initial ILT practice [*t*_(126)_ = 4.93, *p* = 0.426]. A parametric Mann-Whitney test indicated similar findings with a non-significant group difference at ILT testing (*Z* = 1.31, *p* = 0.197), but a significant difference at the transfer test (*Z* = 2.67, *p* = 0.023), in similar to the significant difference found at initial testing (*Z* = 2.32, *p* = 0.020).

##### Erroneous shapes

The analysis of the ILT erroneous shapes (shapes not produced in one segment *and* that are too narrow or wide with respect to the encircled midpoint) indicated that children with reading and spelling difficulties were less accurate than their peers [*F*_(1, 125)_ = 15.96, *p* < 0.001, η_*p*_^2^ = 0.11; when VMI controlled *F*_(1, 124)_ = 13.92, *p* < 0.001, η_*p*_^2^ = 0.10]. No other main effects or interactions emerged. Much as in previous ILT studies, the accuracy level did not change with practice (Table 3; Adi-Japha et al., 2011b, 2019; Julius and Adi-Japha, 2015). The comparison of transfer condition to retention testing showed only a main effect of reading and spelling difficulties [*F*_(1, 12__5__)_ = 7.08, *p* = 0.01, η_*p*_^2^ = 0.05; when VMI controlled *F*_(1, 124)_ = 6.00, *p* = 0.02, η_*p*_^2^ = 0.05], indicating that children with reading and spelling difficulties were less accurate both at retention testing and at the transfer condition than were their peers.

**TABLE 3:** Analyses of ILT erroneous shapes.

	ILT learning								Transfer task							
	Erroneous shapes				VMI as a covariate				Erroneous shapes				VMI as a covariate			
	DF	*F*	*p*	η^2^	DF	*F*	*p*	η^2^	DF	*F*	*p*	η^2^	DF	*F*	*p*	η^2^
Time-Point	4, 500	0.64	0.635	0.00	4, 496	1.02	0.395	0.01	1, 125	0.14	0.707	0.00	1, 124	5.56	0.020	0.04
Time-Point × VMI					4, 496	0.86	0.490	0.01					1, 124	5.96	0.016	0.05
Time-Point × ME	4, 500	1.09	0.359	0.01	4, 496	1.15	0.330	0.01	1,125	1.61	0.207	0.01	1, 124	2.01	0.159	0.02
Time-Point × reading and spelling difficulties	4, 500	1.62	0.17	0.01	4, 496	1.59	0.176	0.01	1,125	3.23	0.075	0.02	1, 124	2.18	0.142	0.02
Time-Point × ME × reading and spelling difficulties	4, 500	0.78	0.54	0.00	4, 496	0.073	0.573	0.01	1,125	0.24	0.627	0.00	1, 124	0.14	0.705	0.00
VMI					1, 124	3.76	0.055	0.03					1, 124	1.20	0.160	0.02
ME	1, 125	1.21	0.27	0.01	1, 124	1.47	0.227	0.01	1,125	0.83	0.365	0.01	1, 124	0.97	0.326	0.01
Reading and spelling difficulties	1, 125	15.96	0.00	0.11	1, 124	13.92	0.000	0.10	1,125	7.08	0.009	0.05	1, 124	6.00	0.016	0.05
ME × reading and spelling difficulties	1, 125	2.60	0.11	0.02	1, 124	2.36	0.127	0.02	1,125	2.58	0.111	0.02	1, 124	2.38	0.125	0.02

*VMI, Visual Motor Integration raw score; ME, Maternal Education level (low/high); reading and spelling difficulties, Reading and Spelling Difficulties.*

#### Performance Times

**FIGURE 4:**
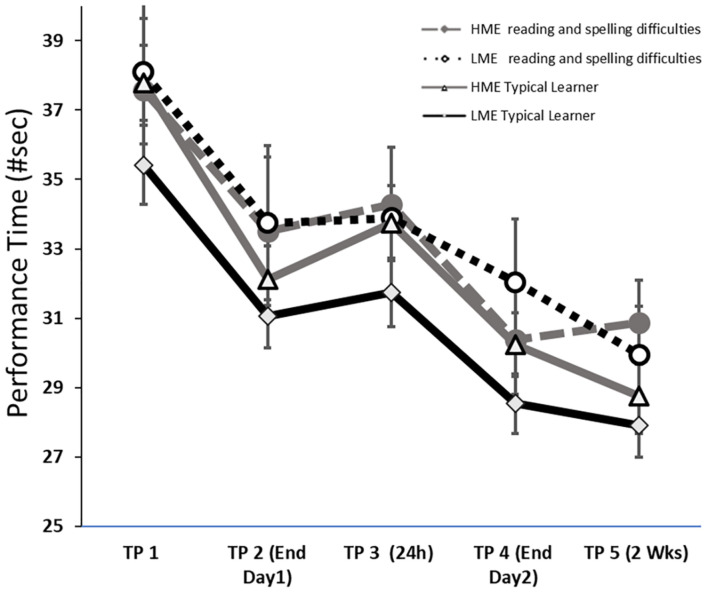
ILT Performance times (#sec, mean and standard deviation) in the four groups: *HME typical learner*, typical learner with high maternal education; *HME typical learner*, typical learner with high maternal education; *LME* typical learner, typical learner with low maternal education; *HME reading and spelling difficulties*, reading and spelling difficulties with high maternal education; *LME reading and spelling difficulties*, reading and spelling difficulties with low maternal education. The figure depicts the five points in time: *TPI (Init Day 1*) Initial Day 1: *TP2 (End Day 1)* end of Day 1, *TP3 (24 h) post-training* initial of Day 2, *TP4 (End Day 2)* end of Day 2; *TP5 (2 Weeks)* and 2 weeks post-training retention testing.

The analysis of ILT performance times (Figure 4) found an overall improvement only [*F*_(3.0__7__, 387.66)_ = 53.30, *p* < 0.001, η_*p*_^2^ = 0.30]. Pairwise comparison indicated improvement during Day 1 (*p* < 0.001), during Day 2 (*p* < 0.001), and from end of Day 2 to 2 weeks post-training (*p* = 0.049). When VMI scores were controlled, the overall improvement became non-significant (see Table 4). In the comparison of transfer condition to retention testing, neither main effects nor interactions were found (Table 4).

**TABLE 4:** Analyses of ILT completion times.

	ILT learning								Transfer task							
	Time				VMI as a covariate				Time				VMI as a covariate			
	DF	*F*	*p*	η^2^	DF	*F*	*p*	η^2^	DF	*F*	*p*	η^2^	DF	*F*	*p*	η^2^
Time-Point	4, 500	53.30	0.000	0.30	4, 496	0.53	0.714	0.00	1, 125	11.77	0.001	0.09	1, 124	1.42	0.235	0.01
Time-Point × VMI					4, 496	2.29	0.059	0.02					1, 124	2.91	0.090	0.02
Time-Point × ME	4, 500	0.26	0.903	0.00	4, 496	0.25	0.913	0.00	1, 125	0.01	0.929	0.00	1, 124	0.00	0.999	0.00
Time-Point × reading and spelling difficulties	4, 500	0.22	0.925	0.00	4, 496	0.17	0.954	0.00	1, 125	0.00	0.961	0.00	1, 124	0.08	0.775	0.00
Time-Point × ME × reading and spelling difficulties	4, 500	0.60	0.662	0.00	4, 496	0.58	0.680	0.00	1, 125	1.06	0.306	0.01	1, 124	1.24	0.267	0.01
VMI					1, 124	0.20	0.656	0.00					1, 124	4.10	0.045	0.03
ME	1, 125	1, 124	0.686	0.00	1, 124	0.18	0.671	0.00	1, 125	0.27	0.607	0.00	1, 124	0.39	0.531	0.00
Reading and spelling difficulties	1, 125	1, 124	0.183	0.01	1, 124	1.59	0.210	0.01	1, 125	2.87	0.093	0.02	1, 124	2.01	0.159	0.02
ME × reading and spelling difficulties	1, 125	1, 124	0.653	0.00	1, 124	0.22	0.639	0.00	1, 125	0.01	0.915	0.00	1, 124	0.00	0.991	0.00

*VMI, Visual Motor Integration raw score; ME, Maternal Education level (low/high); reading and spelling difficulties, Reading and Spelling Difficulties.*

### Verbal Learning

#### Accuracy Performance – Overall, Initial and Benefits of Practice

**FIGURE 5:**
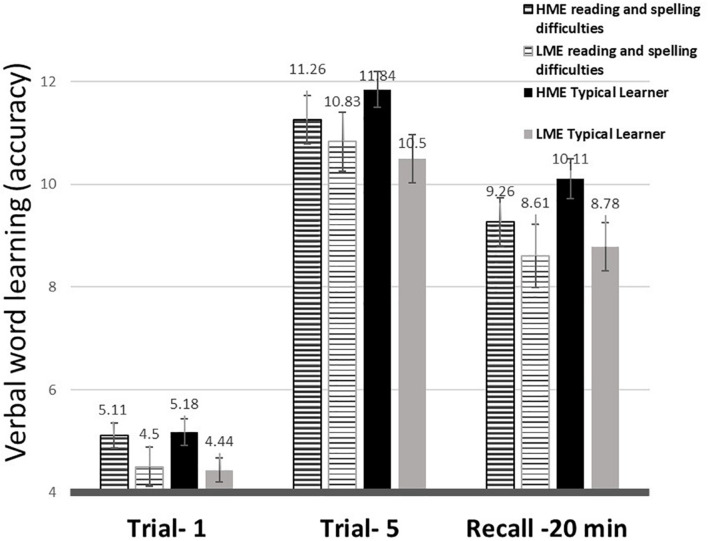
*Verbal word-learning (accuracy*, mean and standard deviation*)* trajectory in the four groups: *HME reading and spelling difficulties*, reading and spelling difficulties with high maternal education; *LME reading and spelling difficulties*, reading and spelling difficulties with low maternal education.

A 3 (time-points = RAVLT 1, RAVLT 5, RAVLT at 20-min lag) × 2 (ME = low/high) × 2 (reading and spelling difficulties = with/without) mixed-design ANOVA was used to study the effects of ME and reading and spelling difficulties on verbal learning scores. The analysis revealed an overall Time-point main effect [*F*_(1.72, 12__5__)_ = 328.99, *p* < 0.001, η_*p*_^2^ = 0.73], with increase of knowledge with repetitions, but decrease at recall (*p*’s < 0.001). The analysis further indicated that children of mothers with a higher education overall recalled more at all the study phases [*F*_(1,12__5__)_ = 5.70, *p* = 0.02, η_*p*_^2^ = 0.04] (see Figure 5 and Table 5). To verify that differences between groups did not occur due to differences in short term verbal memory (assessed using the forward digit-span task; Table 1) or lower vocabulary scores, the analysis was repeated, with STM and vocabulary scores used as covariates. This analysis indicated similar effects: an overall Time-point main effect [*F*_(__2_, _246__)_ = 19.34, *p* < 0.001, η_*p*_^2^ = 0.14], with increase of knowledge with repetitions, but decrease at recall (*p*’s < 0.001) and an advantage for children with higher ME [*F*_(1,__12__3__)_ = 3.95, *p* = 0.049, η_*p*_^2^ = 0.03].

**TABLE 5:** Analyses of the verbal word-learning task.

	Rey AVLT learning				Rey AVLT with STM and vocabulary as covariates			
		
	DF	*F*	*p*	η^2^	DF	*F*	*p*	η^2^
Time-Point	2, 250	328.99	0.000	0.73	2, 246	19.34	0.000	0.14
Time-Point × STM					2, 246	1.17	0.311	0.01
Time-Point × Vocabulary					2, 246	1.25	0.289	0.01
Time-Point × ME	2, 250	0.66	0.519	0.01	2, 246	0.40	0.673	0.00
Time-Point × reading and spelling difficulties	2, 250	1.32	0.269	0.01	2, 246	0.95	0.389	0.01
Time-Point × ME × reading and spelling difficulties	2, 250	0.15	0.857	0.00	2, 246	0.10	0.901	0.00
STM					1,123	1.23	0.269	0.01
Vocabulary					1,123	10.12	0.002	0.08
ME	1, 125	5.70	0.020	0.040	1,123	3.95	0.049	0.03
Reading and spelling difficulties	1, 125	0.508	0.477	0.004	1,123	0.02	0.899	0.00
ME × reading and spelling difficulties	1, 125	0.531	0.467	0.004	1,123	0.66	0.419	0.01

*Vocabulary Number of correctly described words of the WISC-R 95 subtest raw score; Verbal STM, Verbal short-term memory – the Number recall subtest of the WISC-R 95 raw score; ME, Maternal Education level (low/high); reading and spelling difficulties, Reading and Spelling Difficulties.*

## Discussion

This study focused on the effect of ME and reading and spelling difficulties on learning by repetition in a sample of second graders from low-SES families. About 25% of the study sample, second-grade students from two primary schools in a low-SES municipality, were children with reading and spelling difficulties. The sample was equally distributed among children with low or higher ME. A grapho-motor skill learning task (the ILT) and a verbal intentional word-learning task (the RAVLT) were used to study children’s learning by repetition. Previous research suggests that children with impaired reading or with low ME may have deficits in visual-motor as well as verbal learning. In addition to identifying atypical learning patterns, we were particularly interested in whether the effects of ME and reading and spelling difficulties are independent (i.e., implying a greater level of difficulty for children with lower ME and reading and spelling difficulties).

In the current study, children learned the motor and verbal task as expected. As for the ILT, the accuracy level, assessed by the occurrences of surplus segments and erroneous letter shapes, was maintained throughout the task. Production times became shorter with practice during the two training days and improved further by 2-weeks post-training indicating consolidation and retention of the skill (Julius and Adi-Japha, 2015). However, in line with the research hypotheses suggesting lower performance level on motor skills in children with lower ME (Valadi and Gabbard, 2020), these children produced overall more surplus segments. In line with the research hypotheses suggesting lower performance levels on motor skills in children with reading and spelling difficulties (Downing and Caravolas, 2020), children with reading and spelling difficulties produced overall more erroneous shapes. Moreover, children with reading and spelling difficulties had more surplus segments 24-h post-initial training (i.e., at the beginning of Day 2) than they produced at the end of Day 1 training. Vicari et al. (2005) studied the grapho-motor version of the mirror drawing task in children with dyslexia and similarly found worse performance 24-h post-training. Together, these results corroborate the notion of poorer (grapho-)motor performance and atypical (grapho-)motor skill learning in children with reading and spelling difficulties.

On the word-learning task, children improved with training and lost some of the words they learned following the 20-min. delay period, as expected for this declarative-intentional task (Vakil et al., 2010). In line with the research hypothesis, children with lower ME had overall lower recall scores on this word-learning task. No interaction with time-point was found, indicating similar ME differences across the task. van Tetering et al. (2018) reported similar findings on a pictorial verbal learning task. Specifically, lower parental education was associated with lower performance across the task. In the current study children with reading and spelling difficulties had similar recall levels as their peers. These results are in line with the finding of low association between dyslexia and verbal short-term/working memory (Landerl et al., 2013).

Overall, the results of the current study suggest that ME and reading and spelling difficulties affected different components in the learning tasks. In particular, children with low ME and with reading and spelling difficulties had overall poorer performance than their peers with low ME but no reading and spelling difficulties (as on the motor task the former tended to have more erroneous shapes, *p* = 0.055); However, these children performed in similar to their peers who had reading and spelling difficulties but higher ME on both the motor and the verbal task.

### Maternal Education Effects

Our findings indicate that children with higher ME carried out the motor skill learning task (the ILT) with a lower number of surplus segments. Gaps in ILT performance between children of higher and lower ME levels were not significant at the 2-week post-training assessment, but re-emerged at the transfer condition assessed at the same time. The addition of surplus segments characterizes the performance of younger children (Julius and Adi-Japha, 2015), suggesting an overall less mature task performance.

Maternal education may be taken as a proxy for SES. Only one study (Leonard et al., 2015) tested the effect of SES on skill learning, using a probabilistic classification and conducted among adolescents (13–15 years). Leonard et al.’s (2015) study found no significant SES difference, either at the overall performance level or due to learning. These results are similar to the findings of the current study regarding no effect of ME on learning. Differences in overall performance level may have emerged here due to overall lower motor skills in children of mothers with low ME (Dinehart and Manfra, 2013; Africa and van Deventer, 2017; Arrhenius et al., 2018).

Children with higher ME had higher scores on the verbal word-learning task (RAVLT), even when STM and vocabulary scores were used as covariates in the analysis. The declarative-intentional verbal word-learning task involved one session of five learning repetitions followed by a 20-min delayed recall, in which the maternal-education disparities were maintained. Although we were unable to find studies on the effect of SES on verbal learning in children, sustained exposure to poverty and perceived financial difficulty across a 25-year period has been associated with lower performance levels on the RAVLT among adults (Al Hazzouri et al., 2017). Furthermore, 8- to 12-year-olds with higher parental education outperformed their peers on a pictorial word-learning test and, much as in the current study, the differences emerged due to their initial level and did not intensify with practice or at recall (van Tetering et al., 2018).

### Reading and Spelling Difficulties Effects

Children with reading and spelling difficulties differed significantly from their peers in performing the motor skill learning task – the ILT. Overall, these participants were spatially less accurate than were their peers as assessed by the number of erroneous shapes. This may be related to their lower visual-motor skills (Costa et al., 2018), although the group difference remained even when the Beery-VMI scores were used as a covariate. The finding of lower accuracy scores on motor tasks is in line with reports of high prevalence of motor difficulties among individuals with reading and spelling difficulties (Downing and Caravolas, 2020).

Furthermore, 24 h after training, these children produced more segments than required and more than their peers. The finding of lower performance scores on a skill-learning task 24 h after training replicates previous results found among older children with reading disabilities (Vicari et al., 2005; Needle et al., 2006; Hedenius et al., 2013), in particular the findings of Vicari et al. (2005), who also studied a grapho-motor task – the mirror drawing task. The finding of an atypical learning curve 24 h post-training may suggest atypical consolidation processes in this group of reading and spelling difficulties children. These consolidation deficits were later remediated either because of additional task repetitions (insignificant group difference at the end of the second day training session) and/or due to additional time in sleep (insignificant group difference at 2 weeks post-training). The atypical learning pattern in this study was found only for the surplus-segments variable, but not for the more general variable of erroneous shapes. The use of surplus segments is associated with basic production difficulties (e.g., in children with DCD; Adi-Japha and Brestel, 2020), and deficits are more common among younger children (Julius and Adi-Japha, 2015), while the overall accuracy measure of erroneous shapes is taken more as a measure of movement planning using spatial guidelines (Adi-Japha and Brestel, 2020). The use of additional segments following a delay period as a unique measure of deficit was reported for kindergarten children (Julius and Adi-Japha, 2015) and is possibly related to a warm-up decrement at the beginning of a new training session, in line with accounts of a decrease in performance due to forgetting of behavioral support structure for the skill following a delay period (Adams, 1952, 1987; Adi-Japha and Abu-Asba, 2014).

### Study Limitations

The findings presented above must be considered within the limitations of the study. Our participants came from a low-SES city. This casts doubt on our ability to generalize the findings to other population groups. However, we were specifically interested in the population of children with reading and spelling difficulties among the low-SES population. This study needs to be scaled by sampling more socioeconomic variables, such as schools from different districts and the education, occupation, and income of middle- to -high-SES parents. Furthermore, research on verbal learning among low-SES children is currently lacking, and the tools and methods for such inquiry need to be expanded. Another, more specific, limitation has to do with the tests used to measure verbal learning. The RAVLT measures declarative-intentional word-learning on a single day with a 20-min interval allowed for recalling the word. Although it is a well-used and well-studied test with high validity and reliability, it does not afford a view of the learning curve of long-term memory consolidation and retention. Future studies should consider expanding this metric. While studying performance of children with learning difficulties, it is common to control for potential differences in specific skills. Here we used task- related measures (e.g., Beery VMI for grapho-motor learning, see also Adi-Japha and Abu-Asba, 2014). However, one may also control for the more general measure of full-scale IQ with non-verbal as well as verbal skills in one score, rather than using several measures. The current study pertains to children at probable risk, those in the lowest achievement quartile. These children, who could be described as low achievers, may be better positioned to improve their reading and spelling scores than children with diagnosed disabilities, thus suggesting that the conclusions drawn from this sample may differ from those deduced regarding children with more severe difficulties. Nevertheless, previous studies have found that low achievers in this age group who improve are not necessarily different from their peers on all language or literacy tests (Spira et al., 2005).

In conclusion, our results demonstrate that maternal education influences performance level in the low-SES population, but does not hold back learning, *per se*. Reading and spelling difficulties, however, were shown here to affect performance accuracy throughout the task *and* the way the task is learned. Twenty-four hours after training, children with reading and spelling difficulties were less accurate and required more segments to perform a simple grapho-motor task than did their peers – a gap that was closed at retention testing, once the skill was learned and became automatic, 2 weeks later. These findings suggest that young children in low-SES environments, at probable risk of reading and spelling difficulties, might have an atypical learning pattern, and therefore, need individually tailored learning programs and appropriate guidance and support. Importantly, ME and reading and spelling difficulties had specific effects – with ME effects being more diverse (affecting both tasks), thus requiring early support. Finally, this study contributes to the growing literature about children’s learning patterns and calls for future studies that will promote the use of assessments through repetitive learning tasks.

### Theoretical Aspects

One main objective of the study was to find associations between skill-learning and literacy acquisition – as evidenced by the presence of reading and spelling difficulties – among children from a low-SES background who have higher or lower ME. Clear association between skill-learning and literacy acquisition is currently lacking, and conflicting findings have been reported (West et al., 2018). The findings reported here corroborate the notion of overall lower motor performance in children with reading and spelling difficulties, especially those with lower ME. Furthermore, the current findings stress an atypical skill learning curve, as assessed using a grapho-motor task, in children with reading and spelling difficulties coming from low-SES backgrounds. The findings suggest that differentiation among learners’ ME backgrounds, can add to previous work in the field (e.g., Ullman, 2001, 2004; Ullman and Pierpont, 2005; Berninger, 2009; Berninger et al., 2012, 2015a, b; Nicolson and Fawcett, 2019; the recent work of Julius and Adi-Japha, 2015, 2016; and others).

### Practical Aspects

The children included in this study came from a relatively disadvantaged population and were defined as typical learners or at risk of literacy disorders. The latter may have been diagnosed as children with specific learning disorders (SLDs) later on. Children from disadvantaged populations may have a lower initial starting point in terms of declarative memory (Leonard et al., 2015), verbal capabilities (Spira et al., 2005), and fine motor abilities (Arrhenius et al., 2018), as this study has verified. The findings suggest that children from disadvantaged populations – and in particular those among them who have mothers with a lower education level – require early support both in the linguistic and in the motor domain. This may be achieved through age-appropriate play activities (e.g., Adi-Japha and Freeman, 2000; Freeman and Adi-Japha, 2008; Adi-Japha et al., 2010; Bloma et al., 2021). Implications for children with reading and spelling difficulties emerging from this study are that these children require ongoing support during school learning not only for literacy learning but also when learning motor-related tasks. Finally, this work contributes to the growing literature about the learning patterns of children from low-SES backgrounds, calling for future studies on subpopulations within this heterogeneous population.

## Data Availability Statement

The original contributions presented in the study are included in the article/Supplementary Material, further inquiries can be directed to the corresponding author/s.

## Ethics Statement

The studies involving human participants were reviewed and approved by the Israel Ministry of Education (7883/054 10.32) and parents signed the Ministry of Education consent forms. Written informed consent to participate in this study was provided by the participants’ legal guardian/next of kin.

## Author Contributions

CH collected the data, made initial analyzes and wrote the first draft of the MS under the supervision of EA-J. EA-J conducted the review and editing processes, led the final data analyses and MS final version writing. Both authors contributed to the article and approved the submitted version.

## Conflict of Interest

The authors declare that the research was conducted in the absence of any commercial or financial relationships that could be construed as a potential conflict of interest.

## Publisher’s Note

All claims expressed in this article are solely those of the authors and do not necessarily represent those of their affiliated organizations, or those of the publisher, the editors and the reviewers. Any product that may be evaluated in this article, or claim that may be made by its manufacturer, is not guaranteed or endorsed by the publisher.

## Supplementary Material

Supplementary Figure 1 Wacom tablet placed on a slanted ergonomic board.

Supplementary Figure 2 An ILT block printed on a half A4 piece of paper placed on the slanted tablet.

Supplementary Figure 3 The ILTs’ computer program screen collecting data at action.

Supplementary Table 1 Means and standard deviations of main study measures.

## References

[B1] Adams J. A. (1952). Warm-up decrement in performance on the pursuit-rotor. *Am. J. Psychol.* 65 404–414. 10.2307/1418761 12976564

[B2] Adams J. A. (1987). Historical review and appraisal of research on the learning, retention, and transfer of human motor skills. *Psychol. Bull.* 101 41–74. 10.1037/0033-2909.101.1.41

[B3] Adi-Japha E. Abu-Asba H. (2014). Learning, forgetting, and relearning: skill learning in children with language impairment. *Am. J. Speech Lang. Pathol.* 23 696–707. 10.1044/2014_AJSLP-13-0031 25215440

[B4] Adi-Japha E. Brestel G. (2020). Motor skill learning with impaired transfer by children with developmental coordination disorder. *Res. Dev. Disabil.* 103:103671. 10.1016/j.ridd.2020.103671 32505098

[B5] Adi-Japha E. Freeman N. H. (2000). Regulation and division of labor between cognitive systems controlling action. *Cognition* 76 1–11. 10.1016/S0010-0277(00)00067-6 10822041

[B6] Adi-Japha E. Berberich-Artzi J. Libnawi A. (2010). Cognitive flexibility in drawings of bilingual children. *Child Dev.* 81 1356–1366. 10.1111/j.1467-8624.2010.01477.x 20840226

[B7] Adi-Japha E. Berke R. Shaya N. Julius M. S. (2019). Different post-training processes in children’s and adults’ motor skill learning. *PLoS One* 14:e0210658. 10.1371/journal.pone.0210658 30629711 PMC6328138

[B8] Adi-Japha E. Fox O. Karni A. (2011a). Atypical acquisition and atypical expression of memory consolidation gains in a motor skill in young female adults with ADHD. *Res. Dev. Disabil.* 32 1011–1020. 10.1016/j.ridd.2011.01.048 21349685

[B9] Adi-Japha E. Karni A. Parnes A. Loewenschuss I. Vakil E. (2008). A shift in task routines during the learning of a motor skill: group-averaged data may mask critical phases in the individuals’ acquisition of skilled performance. *J. Exp. Psychol.* 34 1544–1551. 10.1037/a0013217 18980413

[B10] Adi-Japha E. Strulovich-Schwartz O. Julius M. (2011b). Delayed motor skill acquisition in kindergarten children with language impairment. *Res. Dev. Disabil.* 32 2963–2971. 10.1016/j.ridd.2011.05.005 21624816

[B11] Africa E. K. van Deventer K. J. (2017). A motor-skills program to enhance visual motor integration of selected pre-school learners. *Early Child Dev. Care* 187 1960–1970. 10.1016/j.ridd.2011.01.048 21349685

[B12] Al Hazzouri A. Z. Elfassy T. Sidney S. Jacobs D. Yaffe K. (2017). Sustained economic hardship and cognitive function: the coronary artery risk development in young adults study. *Am. J. Prev. Med.* 52 1–9. 10.1016/j.amepre.2016.08.009 27692543 PMC5167656

[B13] Alloway T. P. Alloway R. G. (2010). Investigating the predictive roles of working memory and IQ in academic attainment. *J. Exp. Child Psychol.* 106 20–29. 10.1016/j.jecp.2009.11.003 20018296

[B14] Andonova E. (2015). Parental report evidence for toddlers’ grammar and vocabulary in Bulgarian. *First Lang.* 35 126–136. 10.1177/0142723715574399

[B15] Arrhenius B. Gyllenberg D. Chudal R. Lehti V. Sucksdorff M. Sourander O. (2018). Social risk factors for speech, scholastic and coordination disorders: a nationwide register-based study. *BMC Public Health* 18:739. 10.1186/s12889-018-5650-z 29902994 PMC6002992

[B16] Bardid F. Deconinck F. J. A. Descamps S. Verhoeven L. De Pooter G. Lenoir M. (2013). The effectiveness of a fundamental motor skill intervention in pre-schoolers with motor problems depends on gender but not environmental context. *Res. Dev. Disabil.* 34 4571–4581. 10.1016/j.ridd.2013.09.035 24183475

[B17] Beery K. E. Beery N. A. (2010). *The Beery-Buktenica Developmental Test of Visual-Motor Integration. Beery VMI: With Supplemental Developmental Tests of Visual Perception and Motor Coordination and Stepping Stones Age Norms From Birth to Age Six. Administration, Scoring, and Teaching Manual.* Bloomington: Pearson. 10.1037/t48947-000

[B18] Berninger V. W. (2009). Highlights of programmatic, interdisciplinary research on writing. *Learn. Disabil. Res. Pract.* 24 69–80. 10.1111/j.1540-5826.2009.00281.x 19644563 PMC2717633

[B19] Berninger V. W. Richards T. L. Abbott R. D. (2015a). Differential diagnosis of dysgraphia, dyslexia, and OWL LD: behavioral and neuroimaging evidence. *Read. Writ.* 28 1119–1153. 10.1007/s11145-015-9565-0 26336330 PMC4553247

[B20] Berninger V. W. Swanson H. L. Griffin W. (2015b). “Understanding developmental and learning disabilities within functional-systems frameworks: building on the contributions of JP Das,” in *Cognition, Intelligence, and Achievement*, eds Papadopoulos T. C. Parrila R. K. Kirby J. R. (Amsterdam: Elsevier), 397–418. 10.1016/B978-0-12-410388-7.00019-1

[B21] Berninger V. Richards T. (2010). Inter-relationships among behavioral markers, genes, brain and treatment in dyslexia and dysgraphia. *Future Neurol.* 5 597–617. 10.2217/fnl.10.22 20953351 PMC2953808

[B22] Berninger V. Rijlaarsdam G. Fayol M. (2012). “Mapping research questions about translation to methods, measures, and models,” in *Translation of Thought to Written Text While Composing: Advancing Theory, Knowledge, Methods, and Applications*, eds Fayol M. Alamargot D. Berninger V. W. (Hove: Psychology Press), 27–67.

[B23] Birnbaum J. Geyer C. Kirchberg F. Manios Y. Koletzko B., and ToyBox-study Group. (2017). Effects of a kindergarten-based, family-involved intervention on motor performance ability in 3-to 6-year-old children: the ToyBox-study. *J. Sports Sci.* 35 377–384. 10.1080/02640414.2016.1166390 27033346

[B24] Blicher S. Feingold L. Shany M. (2017). The role of trait anxiety and preoccupation with reading disabilities of children and their mothers in predicting children’s reading comprehension. *J. Learn. Disabil.* 50 309–321. 10.1177/0022219415624101 26869245

[B25] Bloma E. Berke R. Shaya N. Adi-Japha E. (2021). Cognitive flexibility in children with developmental language disorder: drawing of nonexistent objects. *Res. Dev. Disabil.* 93:106137. 10.1016/j.jcomdis.2021.106137 34242844

[B26] Bradley R. H. Corwyn R. F. (2002). Socioeconomic status and child development. *Annu. Rev. Psychol.* 53 371–399. 10.1146/annurev.psych.53.100901.135233 11752490

[B27] Bradley R. H. Corwyn R. F. McAdoo H. P. Coll C. G. (2001). The home environments of children in the United States part I: variations by age, ethnicity, and poverty status. *Child Dev.* 72 1844–1867. 10.1111/1467-8624 11768149

[B28] Brady S. A. (1991). “The role of working memory in reading disability,” in *Phonological processes in literacy: A tribute to Isabelle Y. Liberman*, Brady S. A. Shankweiler D. P. (Mahwah, NJ. Lawrence Erlbaum Associates Publishers), 129–151.

[B29] Brooks-Gunn J. Duncan G. J. (1997). The effects of poverty on children. *Future Child.* 7 55–71. 9299837

[B30] Cadime I. Silva C. Ribeiro I. Viana F. L. (2018). Early lexical development: do day care attendance and maternal education matter? *First Lang.* 38 503–519. 10.1177/0142723718778916

[B31] Carver R. P. (1990). *Reading Rate: A review of Research and Theory.* San Diego, CA: Academic Press.

[B32] Center for Educational Technology (2010). *Arabic is Our Language.* Tel Aviv: Center for Educational Technology.

[B33] Center for Educational Technology (2019). *Magic and Friends.* Tel Aviv: Center for Educational Technology.

[B34] Cheng-Lai A. Li-Tsang C. W. P. Chan A. H. L. Lo A. G. W. (2013). Writing to dictation and handwriting performance among Chinese children with dyslexia: relationships with orthographic knowledge and perceptual-motor skills. *Res. Dev. Disabil.* 34 3372–3383. 10.1016/j.ridd.2013.06.039 23911643

[B35] Chiaravalloti A. Ricci M. Di Biagio D. Filippi L. Martorana A. Schillaci O. (2020). The brain metabolic correlates of the main indices of neuropsychological assessment in Alzheimer’s disease. *J. Pers. Med.* 10:25. 10.3390/jpm10020025 32325686 PMC7354489

[B36] Cohen J. (1988). *Statistical Power Analysis for the Behavioral Sciences.* New York, NY: Erlbaum.

[B37] Compton D. L. Fuchs L. S. Fuchs D. Lambert W. Hamlett C. (2012). The cognitive and academic profiles of reading and mathematics learning disabilities. *J. Learn. Disabil.* 45 79–95. 10.1177/0022219410393012 21444929 PMC3366486

[B38] Costa L. J. Green M. Sideris J. Hooper S. R. (2018). First-grade cognitive predictors of writing disabilities in grades 2 through 4 elementary school students. *J. Learn. Disabil.* 51 351–362. 10.1177/0022219417721182 28720016

[B39] Cotton S. M. Kiely P. M. Crewther D. P. Thomson B. Laycock R. Crewther S. G. (2005). A normative and reliability study for the Raven’s Coloured Progressive Matrices for primary school aged children from Victoria. *Aust. Pers. Ind. Differ.* 39 647–659. 10.1016/j.paid.2005.02.015

[B40] De Bourdeaudhuij I. Van Cauwenberghe E. Spittaels H. Oppert J. M. Rostami C. Brug J. Maes L. (2011). School-based interventions promoting both physical activity and healthy eating in Europe: a systematic review within the HOPE project. *Obes. Rev.* 12 205–216. 10.1111/j.1467-789X.2009.00711.x 20122137

[B41] Dinehart L. Manfra L. (2013). Associations between low-income Children’s fine motor skills in preschool and academic performance in second grade. *Early Educ. Dev.* 24 138–161. 10.1080/10409289.2011.636729

[B42] Dorfberger S. Adi-Japha E. Karni A. (2007). Reduced susceptibility to interference in the consolidation of motor memory before adolescence. *PLoS One* 2:e240. 10.1371/journal.pone.0000240 17327907 PMC1800346

[B43] Downing C. Caravolas M. (2020). Prevalence and cognitive profiles of children with comorbid literacy and motor disorders. *Front. Psychol.* 11:573580. 10.3389/fpsyg.2020.573580 33362640 PMC7759613

[B44] Dudai Y. (2004). The neurobiology of consolidations, or, how stable is the engram? *Annu. Rev. Psychol.* 55 51–86. 10.1146/annurev.psych.55.090902.142050 14744210

[B45] Duncan G. J. Magnuson K. Kalil A. Ziol-Guest K. (2012). The importance of early childhood poverty. *Soc. Indic. Res.* 108 87–98. 10.1007/s11205-011-9867-9

[B46] El Elella S. S. A. Tawfik M. A. El Fotoh W. M. M. A. Barseem N. F. (2017). Screening for developmental delay in preschool-aged children using parent-completed ages and stages questionnaires: additional insights into child development. *Postgrad. Med. J.* 93 597–602. 10.1136/postgradmedj-2016-134694 28408725

[B47] Elliott L. (2020). Sources of heterogeneity in the home learning environments of socioeconomically disadvantaged families. *J. Appl. Dev. Psychol.* 70:101190. 10.1016/j.appdev.2020.101190

[B48] Farah M. J. (2017). The neuroscience of socioeconomic status: correlates, causes, and consequences. *Neuron* 96 56–71. 10.1016/j.neuron.2017.08.034 28957676

[B49] Farmer M. Echenne B. Bentourkia M. H. (2016). Study of clinical characteristics in young subjects with developmental coordination disorder. *Brain Dev.* 38 538–547. 10.1016/j.braindev.2015.12.010 26763621

[B50] Faul F. Erdfelder E. Buchner A. Lang A. G. (2009). Statistical power analyses using G^∗^ Power 3.1: tests for correlation and regression analyses. *Behav. Res. Methods* 41 1149–1160. 10.3758/BRM.41.4.1149 19897823

[B51] Fluss J. Ziegler J. C. Warszawski J. Ducot B. Richard G. Billard C. (2009). Poor reading in French elementary school: the interplay of cognitive, behavioral, and socioeconomic factors. *J. Dev. Behav. Pediatr.* 30 206–216. 10.1097/DBP.0b013e3181a7ed6c 19412126

[B52] Fox O. Karni A. Adi-Japha E. (2016). The consolidation of a motor skill in young adults with ADHD: shorter practice can be better. *Res. Dev. Disabil.* 51 135–144. 10.1016/j.ridd.2016.01.014 26826465

[B53] Freeman N. H. Adi-Japha E. (2008). “Pictorial intention, action and interpretation,” in *Drawing and Non-Verbal Intelligence*, eds Vinter A. Lang-Kutner C. (Cambridge: Cambridge University Press), 104–120. 10.1017/CBO9780511489730.006

[B54] Friend A. DeFries J. C. Olson R. K. (2008). Parental education moderates genetic influences on reading disability. *Psychol. Sci.* 19 1124–1130. 10.1111/j.1467-9280.2008.02213.x 19076484 PMC2605635

[B55] Ganzach Y. (2000). Parents’ education, cognitive ability, educational expectations and educational attainment: interactive effects. *Br. J. Educ. Psychol.* 70 419–441. 10.1348/000709900158218 11059120

[B56] Grissmer D. Grimm K. J. Aiyer S. M. Murrah W. M. Steele J. S. (2010). Fine motor skills and early comprehension of the world: two new school readiness indicators. *Dev. Psychol.* 46:1008. 10.1037/a0020104 20822219

[B57] Gullick M. M. Demir-Lira Ö. E. Booth J. R. (2016). Reading skill–fractional anisotropy relationships in visuospatial tracts diverge depending on socioeconomic status. *Dev. Sci.* 19 673–685. 10.1111/desc.12428 27412229 PMC5995108

[B58] Hamrick P. Lum J. A. Ullman M. T. (2018). Child first language and adult second language are both tied to general-purpose learning systems. *Proc. Natl. Acad. Sci. U.S.A.* 115 1487–1492. 10.1073/pnas.1713975115 29378936 PMC5816159

[B59] Hamstra-Bletz L. Blöte A. W. (1993). A longitudinal study on dysgraphic handwriting in primary school. *J. Learn. Disabil.* 26 689–699. 8151209 10.1177/002221949302601007

[B60] Hart B. Risley T. (2003). The early catastrophe: the 30 million word gap. *Am. Educ.* 27 4–9.

[B61] Hatcher P. J. Hulme C. Miles J. N. Carroll J. M. Hatcher J. Gibbs S. Snowling M. J. (2006). Efficacy of small group reading intervention for beginning readers with reading-delay: a randomised controlled trial. *J. Child Psychol. Psychiatry* 47 820–827. 10.1111/j.1469-7610.2005.01559.x 16898996

[B62] He X. Tong S. X. (2017). Quantity matters: children with dyslexia are impaired in a small, but not large, number of exposures during implicit repeated sequence learning. *Am. J. Speech Lang. Pathol.* 26 1080–1091. 10.1044/2017_AJSLP-15-0190 28796861

[B63] Hedenius M. Persson J. Alm P. A. Ullman M. T. Howard J. H. Howard D. V. (2013). Impaired implicit sequence learning in children with developmental dyslexia. *Res. Dev. Disabil.* 34 3924–3935. 10.1016/j.ridd.2013.08.014 24021394

[B64] Hedenius M. Persson J. Tremblay A. Adi-Japha E. Veríssimo J. Dye C. D. (2011). Grammar predicts procedural learning and consolidation deficits in children with specific language impairment. *Res. Dev. Disabil.* 32 2362–2375. 10.1016/j.ridd.2011.07.026 21840165 PMC3191257

[B65] Hoff E. (2003). The specificity of environmental influence: socioeconomic status affects early vocabulary development via maternal speech. *Child Dev.* 74 1368–1378. 10.1111/1467-8624.00612 14552403

[B66] Hoff E. (2013). Interpreting the early language trajectories of children from low-SES and language minority homes: implications for closing achievement gaps. *Dev. Psychol.* 49:4. 10.1037/a0027238 22329382 PMC4061698

[B67] Hollingshead A. B. (1975). “Four factor index of social status,” in *Paper Presented at Unpublished Working Paper, Department of Sociology*, (New Haven, CT: Yale University).

[B68] Julius M. S. Adi-Japha E. (2015). Learning of a simple grapho-motor task by young children and adults: similar acquisition but age-dependent retention. *Front. Psychol.* 6:225. 10.3389/fpsyg.2015.00225 25798120 PMC4350392

[B69] Julius M. S. Adi-Japha E. (2016). A developmental perspective in learning the mirror-drawing task. *Front. Hum. Neurosci.* 10:83. 10.3389/fnhum.2016.00083 26973498 PMC4773595

[B70] Julius M. S. Meir R. Shechter-Nissim Z. Adi-Japha E. (2016). Children’s ability to learn a motor skill is related to handwriting and reading proficiency. *Learn. Individ. Differ.* 51 265–272. 10.1016/j.lindif.2016.08.034

[B71] Keenan P. A. Ricker J. H. Lindamer L. A. Jiron C. C. Jacobson M. W. (1996). Relationship between WAIS-R vocabulary and performance on the California verbal learning test. *Clin. Neuropsychol.* 10 455–458. 10.1080/13854049608406706

[B72] Korat O. (2009). The effect of maternal teaching talk on children’s emergent literacy as a function of type of activity and maternal education level. *J. Appl. Dev. Psychol.* 30 34–42. 10.1016/j.appdev.2008.10.001

[B73] Korat O. Klein P. Segal-Drori O. (2007). Maternal mediation in book reading, home literacy environment, and children’s emergent literacy: a comparison between two social groups. *Read. Writing* 20 361–398. 10.1007/s11145-006-9034-x

[B74] Kramer J. H. Knee K. Delis D. C. (2000). Verbal memory impairments in dyslexia. *Arch. Clin. Neuropsychol.* 15 83–93. 10.1093/arclin/15.1.83 14590570

[B75] Krishnan S. Watkins K. E. Bishop D. V. (2017). The effect of recall, reproduction, and restudy on word learning: a pre-registered study. *BMC Psychol.* 5:28. 10.1186/s40359-017-0198-8 28778213 PMC5545031

[B76] Krueger L. E. Salthouse T. A. (2010). Differences in acquisition, not retention, largely contribute to sex differences in multitrial word recall performance. *Pers. Individ. Differ.* 49 768–772. 10.1016/j.paid.2010.06.024 21057656 PMC2968742

[B77] Landerl K. Ramus F. Moll K. Lyytinen H. Leppänen P. H. T. Lohvansuu K. (2013). Predictors of developmental dyslexia in European orthographies with varying complexity. *J. Child Psychol. Psychiatry Allied Discip.* 54 686–694. 10.1111/jcpp.12029 23227813

[B78] Leonard J. A. Mackey A. P. Finn A. S. Gabrieli J. D. (2015). Differential effects of socioeconomic status on working and procedural memory systems. *Front. Hum. Neurosci.* 9:554. 10.3389/fnhum.2015.00554 26500525 PMC4597101

[B79] Lezak M. D. Howieson D. B. Loring D. W. Fischer J. S. (2004). *Neuropsychological Assessment.* New York, NY: Oxford University Press.

[B80] Lum J. A. Conti-Ramsden G. Page D. Ullman M. T. (2012). Working, declarative and procedural memory in specific language impairment. *Cortex* 48 1138–1154. 10.1016/j.cortex.2011.06.001 21774923 PMC3664921

[B81] Manfra L. Squires C. Dinehart L. H. Bleiker C. Hartman S. C. Winsler A. (2017). Preschool writing and premathematics predict Grade 3 achievement for low-income, ethnically diverse children. *J. Educ. Res.* 110 528–537. 10.1080/00220671.2016.1145095

[B82] Martínez-García C. Afonso O. Cuetos F. Suárez-Coalla P. (2020). Handwriting production in Spanish children with dyslexia: spelling or motor difficulties?. *Read. Writ.* 34 565–593. 10.1007/s11145-020-10082-w

[B83] Marulis L. M. Neuman S. B. (2010). The effects of vocabulary intervention on young children’s word learning: a meta-analysis. *Rev. Educ. Res.* 80 300–335. 10.3102/0034654310377087

[B84] Mascheretti S. Bureau A. Battaglia M. Simone D. Quadrelli E. Croteau J. (2013). An assessment of gene-by-environment interactions in developmental dyslexia-related phenotypes. *Genes Brain Behav.* 12 47–55. 10.1111/gbb.12000 23176554

[B85] Mcgillion M. Pine J. M. Herbert J. S. Matthews D. (2017). A randomised controlled trial to test the effect of promoting caregiver contingent talk on language development in infants from diverse socioeconomic status backgrounds. *J. Child Psychol. Psychiatry* 58 1122–1131. 10.1111/jcpp.12725 28429816

[B86] McNeill L. H. Kreuter M. W. Subramanian S. V. (2006). Social environment and physical activity: a review of concepts and evidence. *Soc. Sci. Med.* 63 1011–1022. 10.1016/j.socscimed.2006.03.012 16650513

[B87] Mendive S. Lissi M. R. Bakeman R. Reyes A. (2017). Beyond mother education: maternal practices as predictors of early literacy development in Chilean children from low-SES households. *Early Educ. Dev.* 28 167–181. 10.1080/10409289.2016.119701

[B88] Milne A. Plourde L. A. (2006). Factors of a low-SES household: what aids academic achievement? *J. Instr. Psychol.* 33 183–194.

[B89] Morris R. D. Lovett M. W. Wolf M. Sevcik R. A. Steinbach K. A. Frijters J. C. (2012). Multiple-component remediation for developmental reading disabilities: IQ, socioeconomic status, and race as factors in remedial outcome. *J. Learn. Disabil.* 45 99–127. 10.1177/0022219409355472 20445204 PMC9872281

[B90] Needle J. L. Fawcett A. J. Nicolson R. I. (2006). Balance and dyslexia: an investigation of adults’ abilities. *Eur. J. Cogn. Psychol.* 18 909–936. 10.1080/09541440500412304

[B91] Nicolson R. I. Fawcett A. J. (2019). Development of dyslexia: the delayed neural commitment framework. *Front. Behav. Neurosci.* 13:112. 10.3389/fnbeh.2019.00112 31178705 PMC6536918

[B92] Noble K. G. Wolmetz M. E. Ochs L. G. Farah M. J. McCandliss B. D. (2006). Brain–behavior relationships in reading acquisition are modulated by socioeconomic factors. *Dev. Sci.* 9 642–654. 10.1111/j.1467-7687.2006.00542.x 17059461

[B93] OECD (2020). *Education at a Glance 2020: OECD Indicators.* Paris: OECD Publishing. 10.1787/69096873-en

[B94] Overvelde A. Hulstijn W. (2011). Handwriting development in grade 2 and grade 3 primary school children with normal, at risk, or dysgraphic characteristics. *Res. Dev. Disabil.* 32 540–548. 10.1016/j.ridd.2010.12.027 21269805

[B95] Oyler J. D. Obrzut J. E. Asbjornsen A. E. (2012). Verbal learning and memory functions in adolescents with reading disabilities. *Learn. Disabil. Q.* 35 184–195. 10.1177/0731948712436815

[B96] Parush S. Lifshitz N. Yochman A. Weintraub N. (2010). Relationships between handwriting components and underlying perceptual-motor functions among students during copying and dictation tasks. *Occup. Particip. Health* 30 39–48. 10.3928/15394492-20091214-06

[B97] Peterson R. L. Pennington B. F. (2015). Developmental dyslexia. *Annu. Rev. Clin. Psychol.* 11 9.1–9.25. 10.1146/annurev-clinpsy-032814-112842 25594880

[B98] RAMA (2016). The National Authority for Measurement and Evaluation in Education. Mivkhanim be’ivrit le-kita-alef / kita bet [Hebrew Tests for First Grade and Second Grade]. Available online : http://cms.education.gov.il/EducationCMS/Units/Rama/MivchaneyMadafLamore02/SfatEmLekiteAlefBet/IvritAB.htm

[B99] Raven J. Rust J. Squire A. (2008). *Manual: Standard Progressive Matrices Plus and Mill Hill Vocabulary Scale.* London: NCS Pearson.

[B100] Raviv L. Arnon I. (2018). The developmental trajectory of children’s auditory and visual statistical learning abilities: modality-based differences in the effect of age. *Dev. Sci.* 21:e12593. 10.1111/desc.125 28901038

[B101] Rey A. (1964). *The Clinical Examination in Psychology.* Paris: Presses Universitaires de France.

[B102] Romeo R. R. Christodoulou J. A. Halverson K. K. Murtagh J. Cyr A. B. Schimmel C. (2018). Socioeconomic status and reading disability: Neuroanatomy and plasticity in response to intervention. *Cereb. Cortex* 28 2297–2312. 10.1093/cercor/bhx131 28591795 PMC5998958

[B103] Roy P. Chiat S. Dodd B. (2014). *Language and Socioeconomic Disadvantage: From Research to Practice.* London: City University London.

[B104] Savion-Lemieux T. Bailey J. A. Penhune V. B. (2009). Developmental contributions to motor sequence learning. *Exp. Brain Res.* 195 293–306. 10.1007/s00221-009-1786-5 19363605

[B105] Scarborough H. S. (1998). Predicting the future achievement of second graders with reading disabilities: contributions of phonemic awareness, verbal memory, rapid naming, and IQ. *Ann. Dyslexia* 48 115–136. 10.1007/s11881-998-0006-5

[B106] Shany M. Share D. L. (2011). Subtypes of reading disability in a shallow orthography: a double dissociation between accuracy-disabled and rate-disabled readers of Hebrew. *Ann. Dyslexia* 61 64–84. 10.1007/s11881-010-0047-4 21108026

[B107] Shany M. Lachman D. Shalem Z. Bahat A. Zieger T. (2003). *“Ma’akav”—Current Assessment for the Diagnosis of Reading and Writing Disabilities, Based on National Israeli Norms.* Tel Aviv: Yesod Publishing.

[B108] Share D. L. Levin I. (1999). “Learning to read and write in Hebrew,” in *Learning to Read and Write: A Cross-Linguistic Perspective*, (eds) Hatano G. Harris M (Cambridge: Cambridge University Press), 89-111.

[B109] Spira E. G. Bracken S. S. Fischel J. E. (2005). Predicting improvement after first-grade reading difficulties: the effects of oral language, emergent literacy, and behavior skills. *Dev. Psychol.* 41:225. 10.1037/0012-1649.41.1.225 15656751

[B110] Squire L. R. Dede A. J. (2015). Conscious and unconscious memory systems. *Cold Spring Harb. Perspect. Biol.* 7:a021667. 10.1101/cshperspect.a021667 25731765 PMC4355270

[B111] Swanson H. L. Harris K. R (Eds.) (2013). *Handbook of Learning Disabilities.* New York, NY: Guilford.

[B112] Tijms J. (2004). Verbal memory and phonological processing in dyslexia. *J. Res. Readi.* 27 300–310. 10.1111/j.1467-9817.2004.00233.x

[B113] Torgesen J. K. Mathes P. (1999). “What every teacher should know about phonological awareness,” in *Reading Research: Anthology: The Why? of Reading Instruction*, eds Honig B. Diamond L. Nathan R. (Florida: Department of Education Division of Public Schools and Community Education Bureau of Instructional Support and Community Services), 54–61.

[B114] Tsur V. (2006). *Dizzy from A to Z.* Ra’anana: Eric Cohen Books Ltd.

[B115] Ullman M. T. (2001). The neural basis of lexicon and grammar in first and second language: the declarative/procedural model. *Biling. Lang. Cogn.* 4 105–122. 10.1017/S1366728901000220

[B116] Ullman M. T. (2004). Contributions of memory circuits to language: the declarative/procedural model. *Cognition* 92 231–270. 10.1016/j.cognition.2003.10.008 15037131

[B117] Ullman M. T. Pierpont E. I. (2005). Specific language impairment is not specific to language: the procedural deficit hypothesis. *Cortex* 41 399–433. 10.1016/S0010-9452(08)70276-4 15871604

[B118] Ursache A. Noble K. G. (2016). Neurocognitive development in socioeconomic context: multiple mechanisms and implications for measuring socioeconomic status. *Psychophysiology* 53 71–82. 10.1111/psyp.12547 26681619 PMC4685721

[B119] Vakil E. Blachstein H. Wertman-Elad R. Greenstein Y. (2012). Verbal learning and memory as measured by the rey-auditory verbal learning test: ADHD with and without learning disabilities. *Child Neuropsychol.* 18 449–466. 10.1080/09297049.2011.613816 21962025

[B120] Vakil E. Greenstein Y. Blachstein H. (2010). Normative data for composite scores for children and adults derived from the rey auditory verbal learning test. *Clin. Neuropsychol.* 24 662–677. 10.1080/13854040903493522 20155574

[B121] Valadi S. Gabbard C. (2020). The effect of affordances in the home environment on children’s fine-and gross motor skills. *Early Child Dev. Care* 190 1225–1232. 10.1080/03004430.2018.1526791

[B122] van den Burg W. Kingma A. (1999). Performance of 225 Dutch school children on Rey’s Auditory Verbal Learning Test (AVLT): parallel test-retest reliabilities with an interval of 3 months and normative data. *Arch. Clin. Neuropsychol.* 14 545–559. 10.1093/arclin/14.6.545 14590582

[B123] van Strien J. W. (1999). Verbal learning in boys with P-type dyslexia, L-type dyslexia, and boys without learning disabilities: differences in learning curves and in serial position curves. *Child Neuropsychol.* 5 145–153. 10.1076/chin.5.3.145.7336

[B124] van Tetering M. A. de Groot R. H. Jolles J. (2018). Boy–Girl differences in pictorial verbal learning in students Aged 8–12 years and the influence of parental education. *Front. Psychol.* 9:1380. 10.3389/fpsyg.2018.01380 30135667 PMC6092633

[B125] Vellutino F. R. Tunmer W. E. Jaccard J. J. Chen R. (2007). Components of reading ability: multivariate evidence for a convergent skills model of reading development. *Sci. Stud. Read.* 11 3–32. 10.1080/10888430709336632

[B126] Vicari S. Finzi A. Menghini D. Marotta L. Baldi S. Petrosini L. (2005). Do children with developmental dyslexia have an implicit learning deficit? *J. Neurol. Neurosurg. Psychiatry* 76 1392–1397. 10.1136/jnnp.2004.061093 16170083 PMC1739378

[B127] Wechsler D. (1998). *Wechsler Scales of Intelligence-R 95: Hebrew Version.* Jerusalem: Ministry of Education.

[B128] West G. Vadillo M. A. Shanks D. R. Hulme C. (2018). The procedural learning deficit hypothesis of language learning disorders: we see some problems. *Dev. Sci.* 21 e12552. 10.1111/desc.12552 28256101 PMC5888158

[B129] Willingham D. B. (1998). A neuropsychological theory of motor skill learning. *Psychol. Rev.* 105:558. 10.1037/0033-295X.105.3.558 9697430

[B130] Yoshikawa H. Aber J. L. Beardslee W. R. (2012). The effects of poverty on the mental, emotional, and behavioral health of children and youth: implications for prevention. *Am. Psychol.* 67:272. 10.1037/a0028 22583341

[B131] Zadeh Z. Y. Farnia F. Ungerleider C. (2010). How home enrichment mediates the relationship between maternal education and children’s achievement in reading and math. *Early Educ. Dev.* 21 568–594. 10.1080/10409280903118424

