# Supplementary figures and images for: Second Graders’ Grapho-Motor Skill Learning and Verbal Learning: The Effects of Socio-Educational Factors
Source: Front Psychol. 2021 Oct 12;12:687207. doi: 10.3389/fpsyg.2021.687207 (PMC8547519; doi:10.3389/fpsyg.2021.687207)

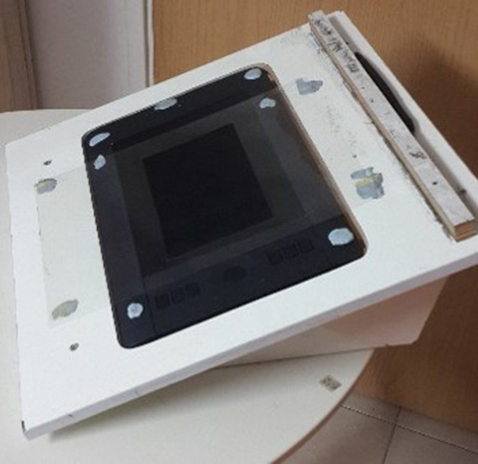

Supplement: Supplementary Figure 1 — Wacom tablet placed on a slanted ergonomic board. [file Image_1.jpeg]

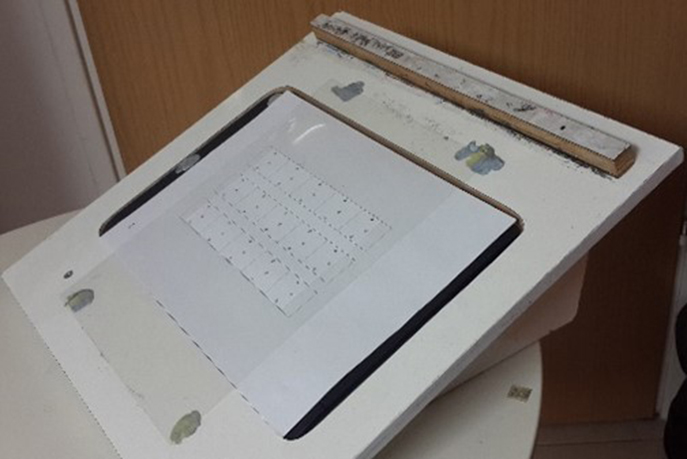

Supplement: Supplementary Figure 2 — An ILT block printed on a half A4 piece of paper placed on the slanted tablet. [file Image_2.jpeg]

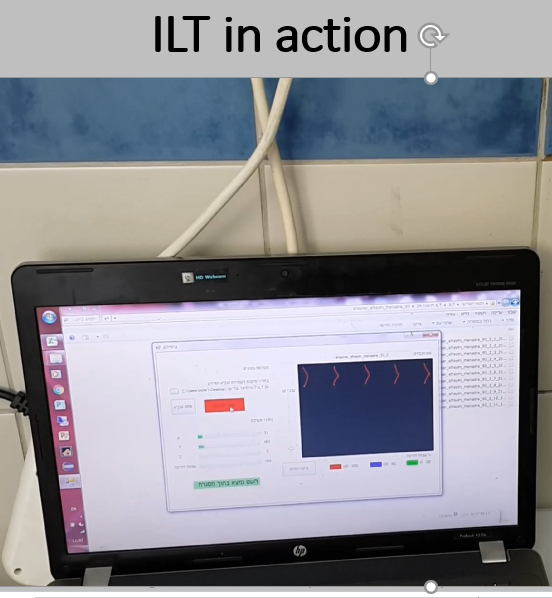

Supplement: Supplementary Figure 3 — The ILTs’ computer program screen collecting data at action. [file Image_3.png]
