# Supplementary material for: Second Graders’ Grapho-Motor Skill Learning and Verbal Learning: The Effects of Socio-Educational Factors
Source: Front Psychol. 2021 Oct 12;12:687207. doi: 10.3389/fpsyg.2021.687207 (PMC8547519; doi:10.3389/fpsyg.2021.687207)
Supplement: Supplementary Table 1 — Means and standard deviations of main study measures. [file Table_1.pdf]

Supplementary Material

Table 1S *Means and Standard Deviations of Main Study Measures*

Measure	Time-points (TP)	Typical learners				reading and spelling difficulties			
		LME		HME		LME		HME	
		M	*SD*	*M*	*SD*	*M*	*SD*	*M*	*SD*
Motor Learning ILT (# errors per block)	Day 1 TP 1	1.54	1.03	1.44	1.31	1.66	1.00	2.28	2.16
	Day 1 TP 2	1.29	1.02	1.22	1.21	1.79	1.87	2.62	2.56
	Day 2 (24h) TP 3	1.27	1.04	1.17	1.02	2.05	2.02	2.07	2.73
	Day 2 (24h) TP 4	1.27	0.92	1.11	0.79	2.09	1.67	2.05	1.08
	2 Week TP 5	1.36	1.37	1.28	1.15	1.86	2.15	2.83	2.15
	Transfer	1.70	1.51	1.46	1.40	1.91	1.58	2.45	2.24
Motor learning ILT (accuracy surplus segments)	Day 1 TP 1	16.29	1.03	15.91	0.92	16.25	0.86	15.89	0.70
	Day 1 TP 2	15.93	0.99	15.67	0.59	15.91	0.64	15.69	0.62
	Day 2 (24h) TP 3	15.86	0.76	15.60	0.49	16.24	1.21	16.08	1.12
	Day 2 (24h) TP 4	15.82	0.83	15.57	0.61	16.13	0.74	15.57	0.49
	2 Week TP 5	15.56	0.53	15.53	0.63	15.60	0.56	15.53	0.62
	Transfer	15.71	0.63	15.55	0.55	15.87	0.57	15.55	0.42
	Day 1 TP 1	35.420	8.07	37.79	7.32	38.09	9.01	37.58	8.99

Motor learning ILT	Day 1 TP 2	31.061	6.54	32.12	6.44	33.74	9.68	33.50	9.35
Performance Time	Day 2 (24h) TP 3	31.742	6.95	33.74	7.24	33.89	8.90	34.29	7.15
(#sec)	Day 2 (24h) TP 4	28.535	6.11	30.23	6.18	32.04	7.90	30.38	6.96
	2 Week TP 5	27.910	6.43	28.75	7.29	29.95	6.09	30.88	5.26
	Transfer	26.22	5.54	28.16	6.75	29.01	5.85	29.39	4.43
Verbal learning Total	REY-AVLT test 1	4.41	1.68	5.18	1.76	4.50	1.69	5.11	1.91
15 words, (# word	REY-AVLT test 5	10.51	3.32	11.82	2.34	10.89	2.56	11.21	1.84
recall)	REY-AVLT test 8	8.84	3.29	10.11	2.65	8.61	2.75	9.26	2.13
Temporal order	REY-AVLTtest10	.13	1.00	.14	1.08	-.38	.99	-.21	1.15

Note. ILT=Invented Letter Task*; M*=Mean*; SD*=Standard Deviation*; TP= Time-points; Verbal learning* =REY Auditory Verbal Learning test
